# Interstitial deletion 4p15.32p16.1 and complex chromoplexy in a female proband with severe neurodevelopmental delay, growth failure and dysmorphism

**DOI:** 10.1186/s13039-022-00610-4

**Published:** 2022-08-05

**Authors:** Dong Li, Alanna Strong, Cuiping Hou, Helen Downes, Amanda Barone Pritchard, Pamela Mazzeo, Elaine H. Zackai, Laura K. Conlin, Hakon Hakonarson

**Affiliations:** 1 Center for Applied Genomics, The Children’s Hospital of Philadelphia, Abramson Research Building, Suite 1016I, 3615 Civic Center Boulevard, Philadelphia, PA 19104-4318 USA; 2 Department of Pediatrics, University of Pennsylvania Perelman School of Medicine, Philadelphia, PA USA; 3 Division of Human Genetics, The Children’s Hospital of Philadelphia, Philadelphia, PA USA; 4 Division of Pediatric Genetics, Metabolism and Genomic Medicine, Department of Pediatrics, University of Michigan Health, Ann Arbor, MI USA; 5 Division of General Pediatrics, The Children’s Hospital of Philadelphia, Philadelphia, PA USA; 6 Division of Genomic Diagnostics, Department of Pathology and Laboratory Medicine, The Children’s Hospital of Philadelphia, Philadelphia, PA USA; 7 Department of Pathology and Laboratory Medicine, Perelman School of Medicine, University of Pennsylvania, Philadelphia, PA USA

## Abstract

Complex chromosomal rearrangements involve the restructuring of genetic material within a single chromosome or across multiple chromosomes. These events can cause serious human disease by disrupting coding DNA and gene regulatory elements via deletions, duplications, and structural rearrangements. Here we describe a 5-year-old female with severe developmental delay, dysmorphic features, multi-suture craniosynostosis, and growth failure found to have a complex series of balanced intra- and inter-chromosomal rearrangements involving chromosomes 4, 11, 13, and X. Initial clinical studies were performed by karyotype, chromosomal microarray, and FISH with research-based short-read genome sequencing coupled with sanger sequencing to precisely map her breakpoints to the base pair resolution to understand the molecular basis of her phenotype. Genome analysis revealed two pathogenic deletions at 4p16.1-p15.32 and 4q31.1, accounting for her developmental delay and dysmorphism. We identified over 60 breakpoints, many with blunt ends and limited homology, supporting a role for non-homologous end joining in restructuring and resolution of the seminal chromoplexy event. We propose that the complexity of our patient’s genomic rearrangements with a high number of breakpoints causes dysregulation of gene expression by three-dimensional chromatin interactions or topologically associating domains leading to growth failure and craniosynostosis. Our work supports an important role for genome sequencing in understanding the molecular basis of complex chromosomal rearrangements in human disease.

**Supplementary Information:**

The online version contains supplementary material available at 10.1186/s13039-022-00610-4.

## Introduction

Microdeletions can produce distinct patterns of malformations and symptomatology secondary to haploinsufficiency of one or multiple genes. Interstitial deletion of 4p14 to 4p16.1 causes a recognizable syndrome of mild to severe intellectual disability, hypotonia, and dysmorphic features, including a long face, upslanting palpebral fissures, a large beaked nose, and a thick lower lip with normocephaly and tall, thin body habitus [6, 19, 21]. The 4p14p16.1 deletion syndrome is genetically and phenotypically distinct from the neighboring microdeletion syndrome Wolf-Hirschhorn syndrome (WHS) characterized by dysmorphic features termed the “Greek warrior helmet” appearance, growth failure and severe developmental delay caused by heterozygous deletion of 4p16.3 [1]. In contrast to WHS in which clear candidate genes have been implicated in the phenotype, the causal genes that dictate the 4p14p16.1 deletion phenotype are unknown.

Microdeletions involving dominant disease associated genes can arise de novo or can be inherited from a similarly or more mildly affected parent. Microdeletions occur as an isolated event, derive from an unbalanced chromosome translocation, or arise in association with complex chromosomal rearrangements [24]. While most balanced rearrangements are not associated with a clinical phenotype, the presence of cryptic copy number variation at the breakpoints is seen in approximately 40% of individuals with an abnormal phenotype due to disruption of additional genes and regulatory elements.

Complex chromosomal rearrangements (CCRs) involve a minimum of five breakpoints and in the case of translocations, a minimum of 2 chromosomes. There are many types of CCRs including chromothripsis, chromoplexy, and chromoanasynthesis [24]. Chromothripsis is caused by localized shattering and random reassembly of tens to hundreds of chromosome segments with non-homologous end-joining or microhomology-mediated end-joining, and can lead to multiple deletions [10, 17]. Chromoplexy, involving multiple chromosomes and primarily reported in somatic cells, is believed to arise secondary to aberrant transcription factor binding leading to the formation of two or more chimeric chromosomes [2]. Chromoanasynthesis is thought to be caused by polymerase stalling leading to localized duplications and triplications often accompanied by chromosome translocations [15]. CCRs are believed to arise from the imperfect repair of a single, catastrophic genomic event [2, 15, 17]. These events play critical roles in genome evolution, as well as human disease, including cancer. Of the three classes of CCRs, chromothripsis is most frequently reported in literature.

Here we present a 5-year-old female with severe global developmental delay, growth failure, craniosynostosis and dysmorphic features found by karyotype, microarray and FISH testing to have de novo deletions at 4p16.1-p15.32 (9.76 Mb), 4q31.1 (881 Kb), and 11q22.1 (771 Kb) that likely occurred as part of a complex chromoplexy event involving four chromosomes. Her clinical presentation of severe growth failure and craniosynostosis could not be explained by her microdeletions. We performed research-based short-read paired-end genome sequencing and Sanger sequencing to better characterize her breakpoints and deletions. We identified 62 break sites, suggesting previously unappreciated complexity to her genomic rearrangement. We propose that more advanced sequencing methodologies are an important tool for understanding the phenotypes of complex chromosomal rearrangements.

## Methods

### Chromosome microarray analysis (CMA)

An Illumina Infinium Global Screening Array (GSA; Illumina, San Diego, Calif) was used to obtain research chromosomal microarray analysis (CMA) for the proband and unaffected parents. The PennCNV algorithm [20] was used for CNV calling. Briefly, log R ratios were used to determine the dosage by intensity of signal, and B allele frequency was calculated using genotype clusters per SNP as determined from HapMap sample analysis. Clinical CMA was independently performed using the Illumina 850 k v1.1 array. Resulted CNVs were interpreted for their pathogenicity by using publicly available databases (DECIPHER, ClinVar, and DGV).

### Karyotyping and fluorescent in situ hybridization (FISH)

Karyotypes of the proband and her parents were performed on peripheral blood lymphocytes at a 550-band level according to standard cytogenetic procedures. FISH was performed on the proband’s peripheral blood metaphases using fosmid or BAC probes: G248P84139G12 (4p16.3), G248P80456F5 (4q12), RP11-785J10 (4q27), G248P800327F6 (4q35.2), G248P85837F3 (11q25), G248P81952B12 (13q34), G248P80323F3 (Xq28/Yq12) and centromere probes (alpha satellite) for chromosomes 4 and 11.

### Short-read genome sequencing and structural variations (SVs) analysis

Whole genome sequencing was performed using the Twist Library Preparation Kit (TWIST Bioscience) and Illumina NovaSeq 6000 at the Center for Applied Genomics (CAG) at the Children’s Hospital of Philadelphia (CHOP). Data were quality controlled and analyzed using a custom-built pipeline [13] that incorporates BWA-mem v0.7.12 [14] for alignment and Picard v1.97 for PCR duplication removal. BAM files generated were fed to short-read structural variant callers, including Manta [3] and Wham [11], to capture SVs with default parameters. Similarly, the split and discordant read files were generated by SpeedSeq [5] and provided as inputs to Lumpy [12], another SV calling program.

### Sanger sequencing of breakpoints

Breakpoint PCR primers were designed using primer 3 and amplicons were sequenced with forward and reverse primers following standard protocols. The UCSC BLAT tool was used to analyze the breakpoints.

## Results

### Patient presentation

The proband was naturally conceived to a 28-year-old G3P1- > 2 mother. There was a history of one prior miscarriage at 8 weeks gestation. Pregnancy was complicated by maternal Rh-negative status treated with Rhogam and ventriculomegaly detected on 20-week ultrasound. Follow up fetal MRI and echocardiogram were normal. Intrauterine growth restriction was noted during the third trimester. Noninvasive prenatal testing (NIPT) for Trisomy 13, 18, 21 and sex-chromosome aneuploidy done at 20 + 1 week gestation was normal.

The proband was born via Caesarian section at 36 2/7 weeks gestational age for breech presentation and persistent intrauterine growth restriction. Birth weight was 1295 g (< 1%; Z = − 2.73), length was 38 cm (< 1%; Z = − 3.63), and head circumference was 29.5 cm (4%; Z = − 1.78). APGAR scores were 8 and 9 at one and 5 min of life, respectively. Physical examination was notable for splayed sagittal sutures, a flattened occiput, large posterior fontanel, down-slanting palpebral fissures, low-set and posteriorly rotated ears, a triangular chin, bilateral 2,3 toe syndactyly and mild head lag. The proband developed conjugated hyperbilirubinemia and was noted to have splenomegaly, prompting abdominal ultrasound, which showed a normal liver and enlarged spleen. A metabolic workup was initiated and was non-diagnostic. Chromosomal SNP microarray was ordered and showed three novel interstitial copy number variations involving chromosomes 4 and 11. Follow up G-banding and FISH analysis showed a complex karyotype involving multiple rearrangements between chromosomes 4, 11, 13 and X consistent with a catastrophic genome wide event to be discussed in greater detail below.

The proband was re-evaluated by Clinical Genetics at 27 months of age. Growth parameters were notable for a weight of 6.485 kg (< 1%; Z = − 7.78), a length of 72 cm (< 1%; Z = − 4.31), and a head circumference of 40.5 cm (< 1%; Z = − 4.76). Physical examination was notable for brachycephaly, prominent sagittal sutures, proptosis with inability to fix or follow, blue sclera, thin skin with prominent veins, sparse hair, and severe hypotonia. Clinical course had evolved to include obstructive sleep apnea with nocturnal CPAP dependence improved by tonsillectomy and adenoidectomy, optic atrophy with preserved visual responsiveness, conductive hearing loss status post bilateral myringotomy tube placement, tethered cord status post release, anemia, thrombocytopenia, hepatosplenomegaly, milk protein intolerance, severe failure to thrive with G-tube dependence, severe global developmental delay, and self-injurious behavior. Additionally, she had Kawasaki disease complicated by dilation of the aortic root, ascending aorta, and coronary arteries. Due to proptosis and prominent sagittal sutures, she was evaluated by Plastic Surgery, and was found to have multi-suture craniosynostosis involving the sagittal and coronal sutures.

**Fig. 1:**
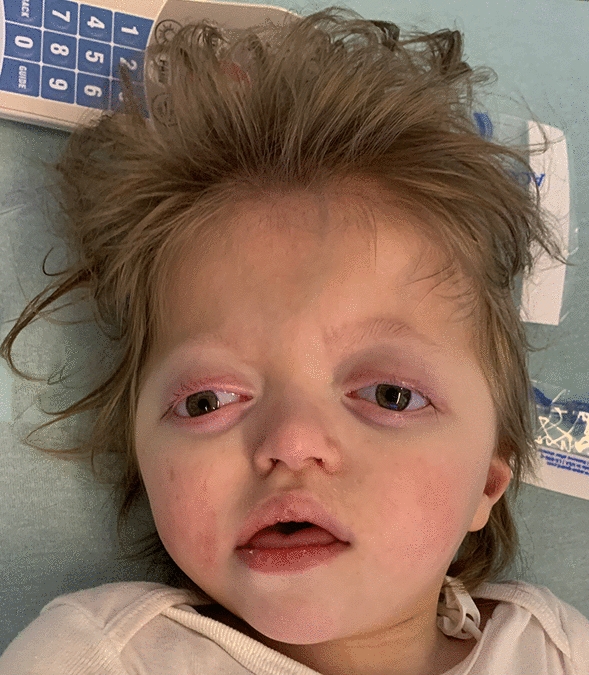
Facial photograph of the studied proband with complex chromosomal rearrangements showing mild proptosis, down-slanting palpebral fissures, and hypertelorism

At 5 years of age, the proband presented with a weight of 12.6 kg (1%; Z = − 3.08), a length of 89.5 cm (< 1%; Z = − 4.08), and a head circumference of 47 cm (< 1%; Z = − 2.39). Physical examination was unchanged, with mild proptosis, down-slanting palpebral fissures, absent fixing and following, hypertelorism, and severe hypotonia (Fig. 1). Her clinical course had evolved to also include chronic constipation with normal barium enema, recurrent C. difficile infection, chronic, patchy inflammatory changes with duodenitis on colonoscopy and esophagogastroduodenoscopy, TPN dependence, post-prandial hypoglycemia with a normal metabolic evaluation, chronic aspiration, oral aversion, chronic otitis media status post bilateral myringotomy tube placement, mild-moderate conductive hearing loss requiring hearing aids, and abnormal EEG showing diffuse and focal cerebral dysfunction with no seizures.

### Clinical molecular testing

**Fig. 2:**
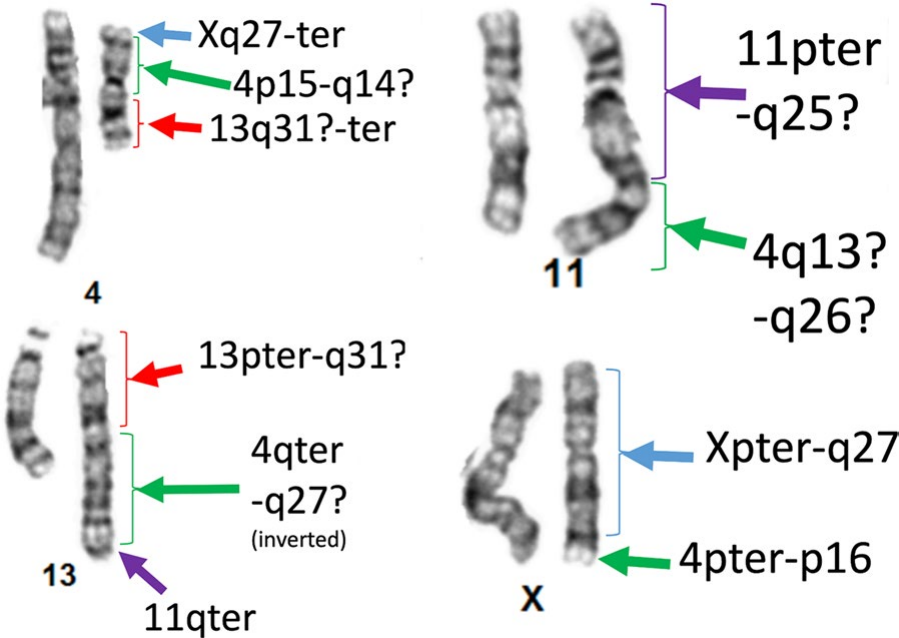
Cytogenetic analysis of chromosomes demonstrating a complex chromosomal rearrangement in 4 chromosomes more consistent with a chromoplexy event

Chromosomal microarray analysis revealed a 9.76 Mb pathogenic deletion within 4p16.1p15.32 [chr4(GRCh37):g.7,145,249–16,906,388] located distal to the Wolf-Hirshhorn critical region at 4p16.3. The microarray analysis also demonstrated an 881 Kb deletion of unknown significance within 4q31.1 [chr4(GRCh37):g.140,115,744–140,996,427], and a 711 Kb deletion of unknown significance within 11q22.1q22.2 [chr11(GRCh37):g.101,524,246–102,235,141] (Additional file 1: Figure S1A). No copy number changes were observed on chromosomes 13 and X (Additional file 1: Figure S1B). Follow-up karyotype and FISH suggested a complex chromosomal rearrangement involving chromosomes 4, 11, 13 and X, consistent with a likely chromoplexy event (Fig. 2). FISH suggested that derivative chromosome 4 consisted of the centromeric region of chromosome 4 with material from Xq replacing 4p and material from 13q replacing 4q. The derivative chromosome 11 contained material from the middle of 4q in place of 11q. The derivative chromosome 13 contained the telomeric region of 4q in inverted orientation as well as the telomeric portion of 11q in place of the 13q telomeric region. The derivative X-chromosome contained the telomeric region of 4p in place of the Xq telomeric region (Fig. 2 and Additional file 1: Figure S2). The karyotype was consistent with 46,X,der(X)(Xpter- > Xq27::4p16.1- > 4pter),der(4)(Xqter- > Xq27::4p15.3- > 4q12::13q31- > 13qter),der(11)(11pter- > 11q25::4q13.1- > 4q26:),der(13)(13pter- > 13q31::4qter- > 4q27::11q25- > 11qter),

### Research genome sequencing

**Fig. 3:**
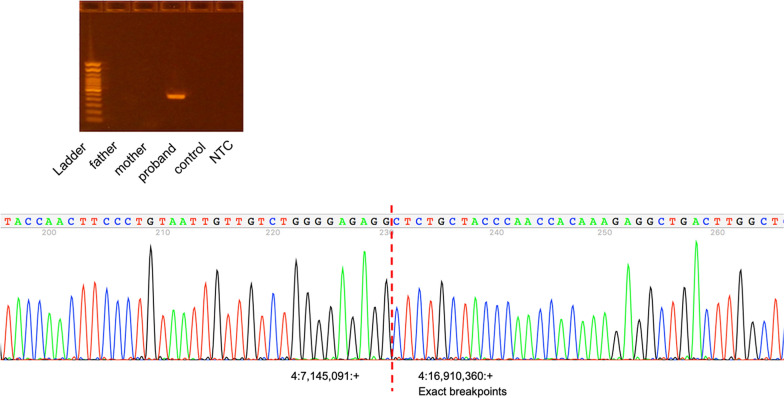
PCR analysis confirmed the de novo pathogenic 9.76 Mb deletion and Sanger sequencing precisely mapped the breakpoints

**Fig. 4:**
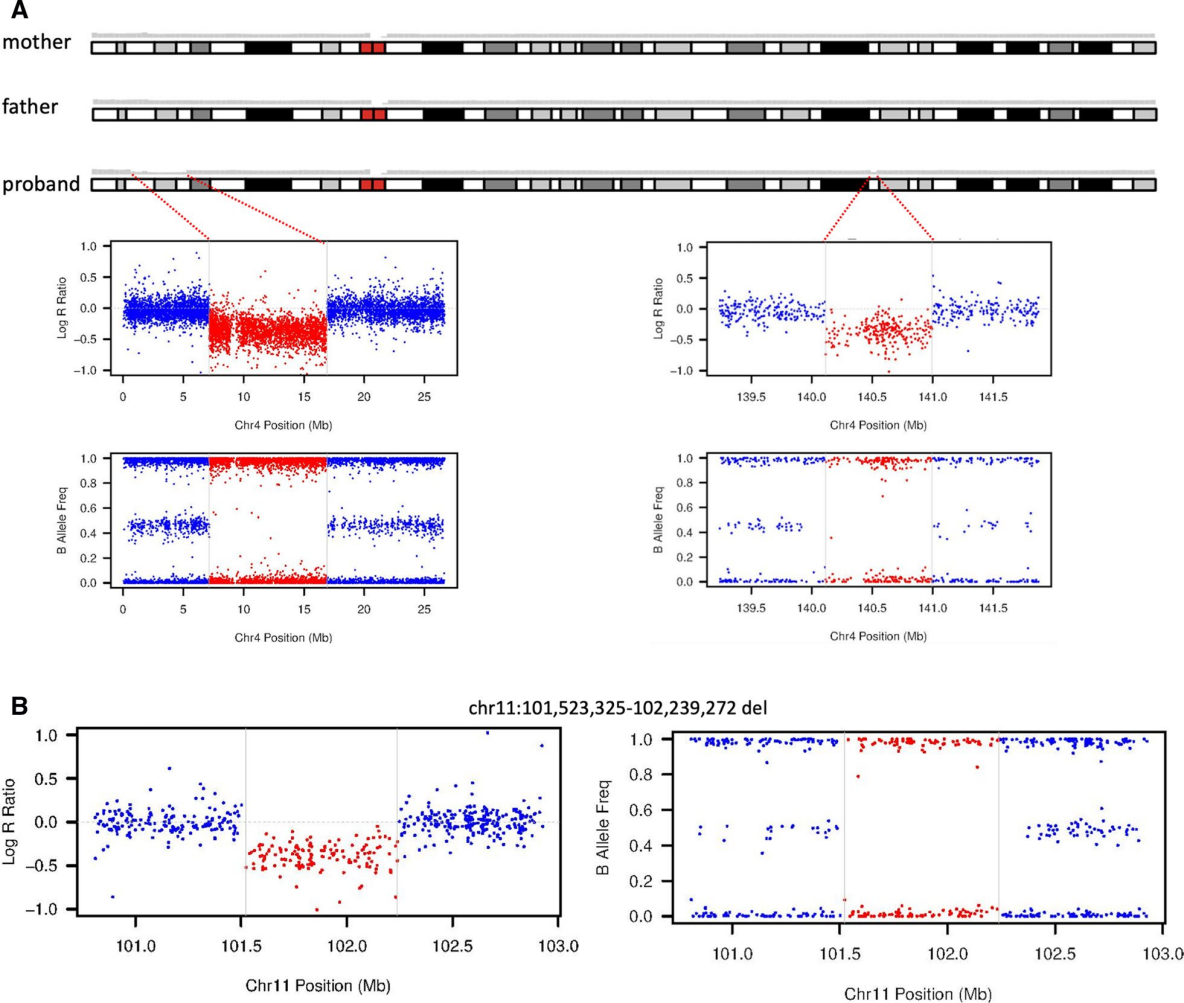
Copy number variation analyses through depth analysis of the whole genome sequencing data and chromosomal microarray analysis. **a** Whole genome depth analysis suggested two de novo deletions that were confirmed by chromosomal microarray analysis. **b** Plot of log R ratio and B allele frequency showing the smaller deletion at 11q22.1q22.2

To map the exact breakpoints of the putative deletions detected by the clinical chromosomal microarray analysis, we performed short-read WGS analysis of the proband and her parents. The average sequencing depth was 28.1x, 26.2x, and 30.2x for proband, mother, and father, respectively. SV calling based on read depth, paired-end read discordance, split-reads, and assembly approaches extended the 9.76 Mb deletion and PCR/Sanger sequencing precisely determined that the deletion is chr4(GRCh37):g.7,145,092–16,910,359 with blunt ends at the breakpoint (Fig. 3). The two smaller deletions of unknown significance at 4q31.1 and 11q22.1q22.2 were not detected by three algorithms (Lumpy, Manta, and Wham). We then manually reviewed the depth of the whole genome level plotted by using kpPlotBAMDensity in an R package karyoploteR. The 9.76 Mb deletion of 4p16.1p15.32 and the deletion at 4q31.1 were evident on the proband’s depth plot (Fig. 4a, upper panel), but not the deletion at 11q22.1q22.2 (data not shown). This prompted us to pursue an independent chromosomal microarray analysis with Illumina Infinium Global Screening Array, which did confirm the two smaller deletions (Fig. 4a, lower panel; and Fig. 4b).

### Breakpoint mapping of complex rearrangements at nucleotide resolution

To obtain detailed insights into the complex rearrangements suggested by the karyotype and FISH analyses and to identify possible genetic candidate(s) for the patient’s unusual clinical phenotype, we comprehensively analyzed the putative de novo structural variations (SVs) from the trio WGS data. SV calling based on multiple approaches suggested six additional deletions, seven duplications, one inversion, six interchromosomal translocations, and 18 one-sided inversion break ends, all de novo in the proband. Interestingly, the majority of these structural rearrangements (5/6 deletions, 7/7 duplications, 1/1 inversion, 6/6 interchromosomal events, and 12/18 one-sided inversion break ends) involved chromosome 4 and overlapped substantially at 4p15 and 4q31q35. Since short-read WGS analysis successfully identified relatively precise breakpoints, PCR/Sanger sequencing was subsequently performed to further clarify these overlapping events. Remarkably, all putative structural rearrangement events, except two (n = 30), were confirmed at the nucleotide level with patient-specific PCR products (Additional file 1: Figure S3 and Additional file 1: Table S1) in 37 successful PCR/Sanger assays, revealing 56 breakpoints in total. Including the six breakpoints generated by the three aforementioned deletions, the patient has 62 breakpoints, with 68% (42/62) of breakpoints affecting chromosome 4 and spanning the entire chromosome, indicating far greater complexity than what was revealed by karyotyping and FISH analyses.

Many of the putative chromosome 4 SVs overlapped substantially, and this is incompatible with the diploid genome. We then reviewed the genome depth plot and alignment for these so-called putative deletions and duplications by the SV calling algorithms and identified no supportive depth drop or evidence of gain (data not shown), suggesting that the breakpoints were not forming conventional SVs, but rather indicative of intrachromosomal and interchromosomal recombinations.

All of the 58 Sanger confirmed breakpoints had either blunt ends or 1–4 bp microhomology sequences with two small insertions (1 and 52 bp) (Additional file 1: Figure S3). All of these breakpoints tended not to cluster at a certain location of the affected chromosome, and the majority of rearrangements led to minimal copy number changes, suggesting that breakpoints were resolved through non-homologous end-joining, consistent with the known mechanism of chromoplexy.

We identified 16 protein-coding genes disrupted by at least 1 breakpoint, 8 of which are associated with human disease (Additional file 1: Table S2) Among them, only *SRP72* and *SGMS2* are associated with autosomal dominant inheritance; however, both genes have a pLI score inconsistent with haploinsuffciency, and the associated phenotypes (bone marrow failure syndrome and calvarial doughnut lesions with bone fragility) have no overlap with our proband (Additional file 1: Table S2). Four gene fusions were identified with three of those fused genes being fused in an incompatible orientation, and thereby unlikely to have a stable RNA transcribed, leaving only one candidate with compatible orientation, a *ALKBH8-BIRC2* fusion. The function of such a fusion protein is unknown.

## Discussion

Here we describe a 5-year-old female with severe developmental delay, growth failure, dysmorphic features, and craniosynostosis found by karyotype, microarray, FISH, and genome sequencing to have three microdeletions and multiple intra- and inter-chromosomal recombinations consistent with a catastrophic chromoplexy event involving at least 4 chromosomes. Careful mapping of her breakpoints demonstrated multiple blunt end breaks with small insertions, suggesting that non-homologous end joining was the mechanism underlying the genome breaks, consistent with our understanding of chromoplexy. While many of her translocations are of unclear clinical significance, her interstitial deletion of 4p16.1p15.32 is classified as pathogenic.

Deletions involving the short arm of chromosome 4 result in at least two clinically distinct syndromes: WHS caused by heterozygous deletion of a 165 Kb critical region (minimum) within 4p16.3 characterized by dysmorphisms, seizures, growth failure, and global developmental delay, and interstitial 4p deletion syndrome involving a genomic region proximal to the WHS critical region characterized by distinct dysmorphic features and developmental delay with normal growth parameters. Our patient’s 4p deletion does not overlap with the WHS critical region, but does include the proximal 4p interstitial deletion critical region, and this likely explains some of her dysmorphic features and developmental delay (Table 1). Of note, growth failure and craniosynostosis are not features of the proximal 4p deletion syndrome.

**Table 1:** Phenotypic features of 4p interstitial deletion syndrome compared to our patient with 4p15.32p16.1 deletion

Clinical features	4p interstitial deletion syndrome	4p15.32p16.1 deletion (current patient)
Developmental delays	Mild to severe	Severe
Hypotonia	10/13	Yes, severe
Tall thin body habitus	5/10	Thin and short stature
Microcephaly	2/12	Yes
Large beaked nose	8/12	No
Long face	6/12	No
Upslanting palpebral fissures	5/12	Down-slanting palpebral fissures
Hypertelorism	4/12	Yes
Craniosynostosis	1/13	Multi-suture

To better understand the molecular basis of our patient’s phenotype we performed genome sequencing to more accurately define the breakpoints and rearrangements of genes and regulatory regions. A likely candidate was our patient’s deletion at 4q31.1, which includes seven genes (*MGARP*, *NDUFC1*, *NAA15*, *RAB33B*, *SETD7*, *MGST2*, and *MAML3*). Since the initial clinical array was performed, haploinsufficiency of *NAA15* has been associated with an autosomal dominant Mendelian neurodevelopmental syndrome (MIM 617787) characterized by variable levels of intellectual disability with global developmental delay and autism spectrum disorder [4, 18]. We therefore reclassify the patient’s 4q31 deletion as pathogenic and suggest that it contributes to our patient’s global delays (Table 2).

**Table 2:** Phenotypic features of *NAA15*-related neurodevelopmental syndrome compared to our patient with 4q31.1 deletion

Clinical features	*NAA15*-related neurodevelopmental syndrome	4q31.1 deletion (current patient)
Developmental delays	Always, mild to severe	Severe
Hypotonia	Sometimes	Yes, severe
Microcephaly	Rare	Yes
Seizures	Sometimes	No
Autism	Frequent	Self-injurious behavior
Heart defects	Sometimes	Yes
Hypertelorism	Rare	Yes
Posteriorly rotated ears	Sometimes	Yes
Triangular chin	Frequent	Yes

The patient’s deletion [chr11(GRCh37):g.101,524,246–102,235,141] at 11q22.1q22.2 includes six protein-coding genes (*ANGPTL5*, *CEP126*, *CFAP300*, *YAP1*, *BIRC3*, and *BIRC2*). *YAP1* has been associated with autosomal dominant coloboma with variable penetrance (MIM 120433), and has a pLI score of 1. Associated phenotypes include isolated ocular coloboma with variable penetrance or coloboma with sensorineural hearing loss, cleft lip/palate, and learning difficulties [22]; however, *YAP1* gene deletion has been reported in a healthy subject [23], and review of our internal SNP microarray dataset (> 21,000 individuals) revealed two other healthy subjects harboring heterozygous deletions of the *YAP1* gene (data not shown). Thus, we consider the deletion at 11q22.1q22.2 a variant of uncertain significance.

Our patient has two large, pathogenic deletions, and it is possible that her severe phenotype relates to a compound effect of both variants. Additive effects of multiple genetic hits have been described for ciliopathy syndromes, hearing loss, and neurodevelopmental disorders; however, in most cases phenotypes are exacerbated by dual hits, but they are not altogether novel, as is the case in our patient. We used short-read genome sequencing and extensive Sanger sequencing to identify critical regulatory regions or novel breakpoints to explain our patient’s atypical phenotype and identified greater than 60 breakpoints across 4 chromosomes with multiple gene fusions and complex rearrangements. Though no distinct causal gene was identified, these methodologies established a higher resolution map of our patient’s genomic rearrangements and revealed far greater complexity than that ascertained by clinical testing. The complexity of our patient’s chromoplexy event and its resolution may ultimately be the explanation for her unique phenotype of growth failure and craniosynostosis. For example, genes associated with these phenotypes may be dysregulated without being disrupted by a breakpoint. Structural rearrangements, including complex chromosomal rearrangements, have been shown to dysregulate gene expression through disruption of three-dimensional chromatin interactions, or topologically associating domains [9, 16]. We queried the ENCODE data to assay for three-dimensional CTCF-bound topologically associated domains (TAD) for these 62 breakpoints and identified one breakpoint that occurred in a cis-regulatory element (ccRE; ENCODE accession number EH38E2348238). This ccRE has a maximum CTCF Z score of 4.17, highly suggestive that it has CTCF-bound insulator property. Nearby protein-coding genes include *CDKN2AIP*, *ING2*, *CLDN24*, *WWC2*, *CLDN22*, *RWDD4*, *TRAPPC11*, *STOX2*, *DCTD*, *TENM3*, *ENPP6*, and *IRF2*. Though many of these genes are involved in growth and cell cycle regulation, further evaluation of the possible contribution of this ccRE to the phenotype is needed.

We noted that several genes on chromosomes 4 and 11 have been associated with craniosynostosis, including *FGFR3*, *WDR19*, *PPP3CA*, *SEC24D*, *SOX6*, *ALX4*, and *B3GAT3*. Analysis of the mRNA gene expression or protein levels of these genes in the relevant patient cells compared to control samples in combination with ccRE analysis may be revealing for her craniosynostosis phenotype.

Our study has several limitations. We did not perform PacBio long-read genome sequencing, optical mapping, or mate-pair short-read genome sequencing, which, if combined with molecular cytogenic techniques, may have provided additional delineation of the structure of the derivative chromosomes or identified additional breakpoints. Importantly, previous studies demonstrated that Illumina short-read paired-end sequencing is very comparable to other platforms in terms of number of breakpoints and base pair resolution [7, 8]. Additionally, short-read genome sequencing analysis based on split-read, depth, and assembly methods was unable to identify the small deletions on chromosomes 4 and 11. This was overcome by concurrent use of cytogenetic assays (including FISH and SNP microarray), highlighting the importance of multiple, synergistic approaches when evaluating complex patients and characterizing complex chromosomal aberrations.

In summary, we present a case of a complex genomic rearrangement causing severe neurodevelopmental delays and growth failure in a young child. Our results suggest that the use of short-read paired-end  genome sequencing can help resolve individual breakpoints and better establish the molecular etiology of patient phenotypes.

## Supplementary Information

**Additional file 1** Additonal genomic data.

## Data Availability

All data needed to evaluate the conclusions in the paper are present in the paper and/or the Supplementary Materials. Additional data related to this paper are available upon reasonable request of the corresponding author.

## References

[1] Battaglia A, Carey JC, South ST (2015). Wolf-Hirschhorn syndrome: a review and update. Am J Med Genet C Semin Med Genet.

[2] Berger MF, Lawrence MS, Demichelis F, Drier Y, Cibulskis K, Sivachenko AY, Sboner A, Esgueva R, Pflueger D, Sougnez C, Onofrio R, Carter SL, Park K, Habegger L, Ambrogio L, Fennell T, Parkin M, Saksena G, Voet D, Ramos AH, Pugh TJ, Wilkinson J, Fisher S, Winckler W, Mahan S, Ardlie K, Baldwin J, Simons JW, Kitabayashi N, MacDonald TY, Kantoff PW, Chin L, Gabriel SB, Gerstein MB, Golub TR, Meyerson M, Tewari A, Lander ES, Getz G, Rubin MA, Garraway LA (2011). The genomic complexity of primary human prostate cancer. Nature.

[3] Chen X, Schulz-Trieglaff O, Shaw R, Barnes B, Schlesinger F, Kallberg M, Cox AJ, Kruglyak S, Saunders CT (2016). Manta: rapid detection of structural variants and indels for germline and cancer sequencing applications. Bioinformatics.

[4] Cheng H, Dharmadhikari AV, Varland S, Ma N, Domingo D, Kleyner R, Rope AF, Yoon M, Stray-Pedersen A, Posey JE, Crews SR, Eldomery MK, Akdemir ZC, Lewis AM, Sutton VR, Rosenfeld JA, Conboy E, Agre K, Xia F, Walkiewicz M, Longoni M, High FA, van Slegtenhorst MA, Mancini GMS, Finnila CR, van Haeringen A, den Hollander N, Ruivenkamp C, Naidu S, Mahida S, Palmer EE, Murray L, Lim D, Jayakar P, Parker MJ, Giusto S, Stracuzzi E, Romano C, Beighley JS, Bernier RA, Kury S, Nizon M, Corbett MA, Shaw M, Gardner A, Barnett C, Armstrong R, Kassahn KS, Van Dijck A, Vandeweyer G, Kleefstra T, Schieving J, Jongmans MJ, de Vries BBA, Pfundt R, Kerr B, Rojas SK, Boycott KM, Person R, Willaert R, Eichler EE, Kooy RF, Yang Y, Wu JC, Lupski JR, Arnesen T, Cooper GM, Chung WK, Gecz J, Stessman HAF, Meng L, Lyon GJ (2018). Truncating variants in NAA15 are associated with variable levels of intellectual disability, autism spectrum disorder, and congenital anomalies. Am J Hum Genet.

[5] Chiang C, Layer RM, Faust GG, Lindberg MR, Rose DB, Garrison EP, Marth GT, Quinlan AR, Hall IM (2015). SpeedSeq: ultra-fast personal genome analysis and interpretation. Nat Methods.

[6] Chitayat D, Ruvalcaba RH, Babul R, Teshima IE, Posnick JC, Vekemans MJ, Scarpelli H, Thuline H (1995). Syndrome of proximal interstitial deletion 4p15: report of three cases and review of the literature. Am J Med Genet.

[7] Eisfeldt J, Pettersson M, Petri A, Nilsson D, Feuk L, Lindstrand A (2021). Hybrid sequencing resolves two germline ultra-complex chromosomal rearrangements consisting of 137 breakpoint junctions in a single carrier. Hum Genet.

[8] Eisfeldt J, Pettersson M, Vezzi F, Wincent J, Kaller M, Gruselius J, Nilsson D, Syk Lundberg E, Carvalho CMB, Lindstrand A (2019). Comprehensive structural variation genome map of individuals carrying complex chromosomal rearrangements. PLoS Genet.

[9] Ibrahim DM, Mundlos S (2020). Three-dimensional chromatin in disease: What holds us together and what drives us apart?. Curr Opin Cell Biol.

[10] Kloosterman WP, Guryev V, van Roosmalen M, Duran KJ, de Bruijn E, Bakker SC, Letteboer T, van Nesselrooij B, Hochstenbach R, Poot M, Cuppen E (2011). Chromothripsis as a mechanism driving complex de novo structural rearrangements in the germline. Hum Mol Genet.

[11] Kronenberg ZN, Osborne EJ, Cone KR, Kennedy BJ, Domyan ET, Shapiro MD, Elde NC, Yandell M (2015). Wham: identifying structural variants of biological consequence. PLoS Comput Biol.

[12] Layer RM, Chiang C, Quinlan AR, Hall IM (2014). LUMPY: a probabilistic framework for structural variant discovery. Genome Biol.

[13] Li D, Bupp C, March ME, Hakonarson H, Levine MA (2020). Intragenic deletions of GNAS in pseudohypoparathyroidism type 1A identify a new region affecting methylation of exon A/B. J Clin Endocrinol Metab.

[14] Li H, Durbin R (2009). Fast and accurate short read alignment with Burrows–Wheeler transform. Bioinformatics.

[15] Liu P, Erez A, Nagamani SC, Dhar SU, Kolodziejska KE, Dharmadhikari AV, Cooper ML, Wiszniewska J, Zhang F, Withers MA, Bacino CA, Campos-Acevedo LD, Delgado MR, Freedenberg D, Garnica A, Grebe TA, Hernandez-Almaguer D, Immken L, Lalani SR, McLean SD, Northrup H, Scaglia F, Strathearn L, Trapane P, Kang SH, Patel A, Cheung SW, Hastings PJ, Stankiewicz P, Lupski JR, Bi W (2011). Chromosome catastrophes involve replication mechanisms generating complex genomic rearrangements. Cell.

[16] Lupianez DG, Spielmann M, Mundlos S (2016). Breaking TADs: how alterations of chromatin domains result in disease. Trends Genet.

[17] Stephens PJ, Greenman CD, Fu B, Yang F, Bignell GR, Mudie LJ, Pleasance ED, Lau KW, Beare D, Stebbings LA, McLaren S, Lin ML, McBride DJ, Varela I, Nik-Zainal S, Leroy C, Jia M, Menzies A, Butler AP, Teague JW, Quail MA, Burton J, Swerdlow H, Carter NP, Morsberger LA, Iacobuzio-Donahue C, Follows GA, Green AR, Flanagan AM, Stratton MR, Futreal PA, Campbell PJ (2011). Massive genomic rearrangement acquired in a single catastrophic event during cancer development. Cell.

[18] Stessman HA, Xiong B, Coe BP, Wang T, Hoekzema K, Fenckova M, Kvarnung M, Gerdts J, Trinh S, Cosemans N, Vives L, Lin J, Turner TN, Santen G, Ruivenkamp C, Kriek M, van Haeringen A, Aten E, Friend K, Liebelt J, Barnett C, Haan E, Shaw M, Gecz J, Anderlid BM, Nordgren A, Lindstrand A, Schwartz C, Kooy RF, Vandeweyer G, Helsmoortel C, Romano C, Alberti A, Vinci M, Avola E, Giusto S, Courchesne E, Pramparo T, Pierce K, Nalabolu S, Amaral DG, Scheffer IE, Delatycki MB, Lockhart PJ, Hormozdiari F, Harich B, Castells-Nobau A, Xia K, Peeters H, Nordenskjold M, Schenck A, Bernier RA, Eichler EE (2017). Targeted sequencing identifies 91 neurodevelopmental-disorder risk genes with autism and developmental-disability biases. Nat Genet.

[19] Tonk VS, Jalal SM, Gonzalez J, Kennedy A, Velagaleti GV (2003). Familial interstitial deletion of chromosome 4 (p15.2p16.1). Ann Genet.

[20] Wang K, Li M, Hadley D, Liu R, Glessner J, Grant SF, Hakonarson H, Bucan M (2007). PennCNV: an integrated hidden Markov model designed for high-resolution copy number variation detection in whole-genome SNP genotyping data. Genome Res.

[21] White DM, Pillers DA, Reiss JA, Brown MG, Magenis RE (1995). Interstitial deletions of the short arm of chromosome 4 in patients with a similar combination of multiple minor anomalies and mental retardation. Am J Med Genet.

[22] Williamson KA, Rainger J, Floyd JA, Ansari M, Meynert A, Aldridge KV, Rainger JK, Anderson CA, Moore AT, Hurles ME, Clarke A, van Heyningen V, Verloes A, Taylor MS, Wilkie AO, Consortium UK, Fitzpatrick DR (2014). Heterozygous loss-of-function mutations in YAP1 cause both isolated and syndromic optic fissure closure defects. Am J Hum Genet.

[23] Wong KK, deLeeuw RJ, Dosanjh NS, Kimm LR, Cheng Z, Horsman DE, MacAulay C, Ng RT, Brown CJ, Eichler EE, Lam WL (2007). A comprehensive analysis of common copy-number variations in the human genome. Am J Hum Genet.

[24] Zepeda-Mendoza CJ, Morton CC (2019). The iceberg under water: unexplored complexity of chromoanagenesis in congenital disorders. Am J Hum Genet.

