# Supplementary material for: Interstitial deletion 4p15.32p16.1 and complex chromoplexy in a female proband with severe neurodevelopmental delay, growth failure and dysmorphism
Source: Mol Cytogenet. 2022 Aug 5;15:33. doi: 10.1186/s13039-022-00610-4 (PMC9354344; doi:10.1186/s13039-022-00610-4)
Supplement: Supplementary file 1 — Additional file 1 Additonal genomic data. [file 13039_2022_610_MOESM1_ESM.docx]

**Table S1**. Putative structure variations by short-read genome sequencing.

| SV | # Support reads pair | # Support reads pair in 106 controls samples * | Chr | Start | End | Length | Cytogenetic location |
| --- | --- | --- | --- | --- | --- | --- | --- |
| DEL1 | 19 | 0 | 4 | 7,145,091 | 16,910,359 | -9,765,268 | 4p16.1-p15.32 |
| DUP1 | 7 | 0 | 4 | 19,866,796 | 26,584,132 | 6,717,336 | 4p15.31-p15.2 |
| DEL2 | 6 | 0 | 4 | 27,288,500 | 31,319,464 | -4,030,964 | 4p15.2-p15.1 |
| INV | 17 | 0 | 4 | 28,638,402 | 31,479,076 | 2,840,674 | 4p15.1 |
| DEL3 | 13 | 0 | 4 | 17,035,795 | 57,336,362 | -40,300,567 | 4p15.32-q12 |
| DEL4 | 31 | 0 | 4 | 121,735,489 | 122,034,447 | -298,958 | 4q27 |
| DUP2 | 14 | 0 | 4 | 122,321,816 | 150,934,793 | 28,612,977 | 4q27-q31.23 |
| DEL5 | 10 | 0 | 4 | 150,078,412 | 150,934,794 | -856,382 | 4q32.23 |
| DUP4 | 10 | 0 | 4 | 143,991,939 | 181,195,049 | 37,203,110 | 4q31.21-q34.3 |
| DUP3 | 22 | 0 | 4 | 143,031,785 | 184,365,699 | 41,333,914 | 4q31.21-q35.1 |
| DEL6 | 13 | 0 | 4 | 140,107,307 | 184,365,699 | -44,258,392 | 4q31.1-q35.1 |
| DUP5 | 6 | 0 | 4 | 142,580,842 | 188,456,325 | 45,875,483 | 4q31.21-q35.2 |
| DUP6 | 9 | 0 | 4 | 181,195,049 | 188,571,176 | 7,376,127 | 4q34.3-q35.2 |
| DUP7 | 25 | 0 | 4 | 184,667,017 | 190,687,135 | 6,020,118 | 4q35.1-q35.2 |
| DEL7 | 9 | 0 | 11 | 130,847,271 | 132,306,812 | -1,459,541 | 11q25 |

* Including two unaffected parents

**Table S2**. Protein-coding genes disrupted by complex rearrangements and gene fusions due to rearrangements.

| Genes or fused genes | Compatible orientation | pLI | OMIM phenotype | OMIM inheritance |
| --- | --- | --- | --- | --- |
| SRP72 |  | 0 | Bone marrow failure syndrome 1 | AD |
| PRDM5 |  | 0 | Brittle cornea syndrome 2 | AR |
| OPCML |  | 0.23 | Ovarian cancer | Somatic |
| INPP4B |  | 0.55 | None | NA |
| IL15 |  | 0.71 | None | NA |
| XIAP |  | 0.92 | Lymphoproliferative syndrome | XLR |
| NTM |  | 0.06 | None | NA |
| SGMS2 |  | 0.06 | Calvarial doughnut lesions with bone fragility with or without spondylometaphyseal dysplasia | AD |
| ALKBH8 |  | 0 | Intellectual developmental disorder | AR |
| ALKBH8-BIRC2 | yes | 0 and 0.43 | Intellectual developmental disorder and none | AR and NA |
| APBB2-TENM1 | no | 0.07 and 1 | none and none | NA and NA |
| ELF2-LRBA | no | 0.99 and 0 | None and common variable inmmunodeficiency | NA and AR |
| LEF1-SORBS2 | no | 1 and 0.04 | Sebaceous tumor and noe | Somatic and none |

**Supplementary Figures**

**S1A**


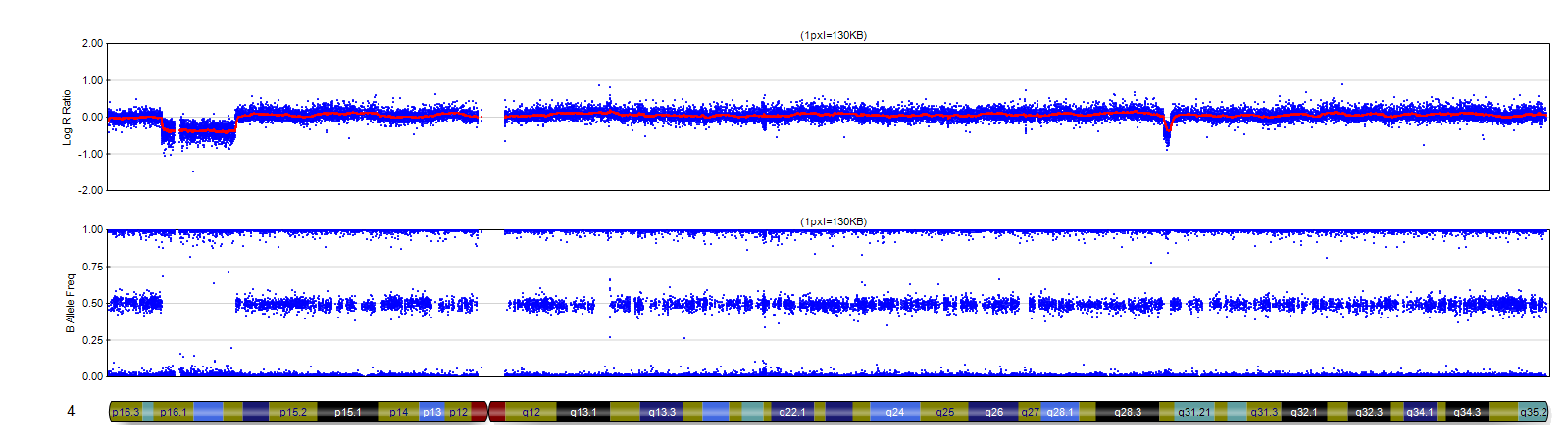


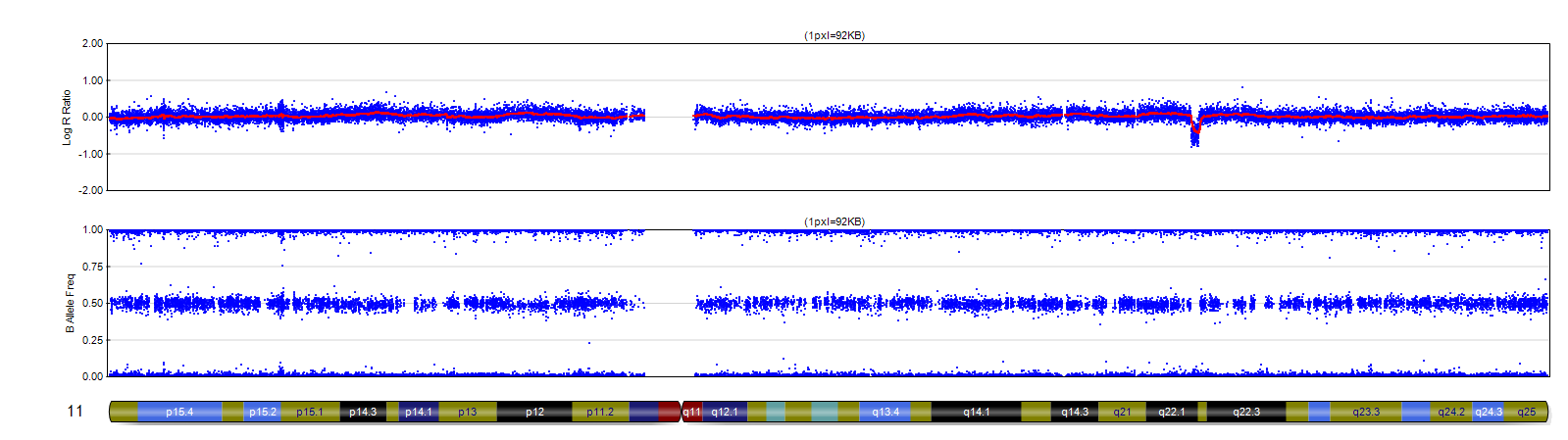


**S1B**


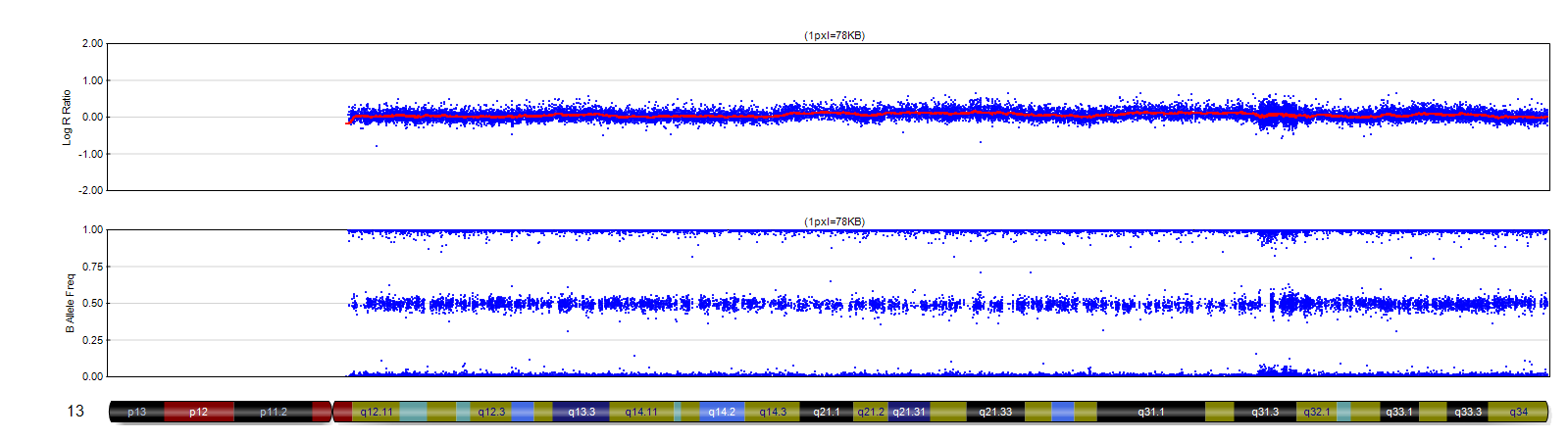


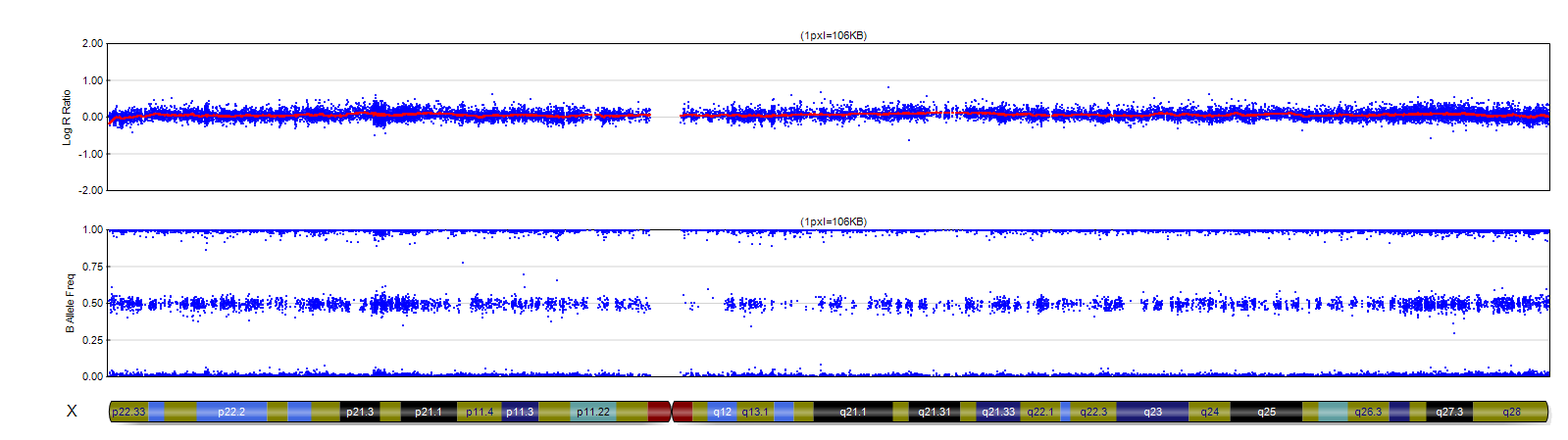


**Figure S1.** **Clinical chromosomal microarray analysis.** (A) The microarray analysis demonstrated two deletions on chromosome 4. (B) No copy number changes were observed on chromosomes 13 and X.

**S2**


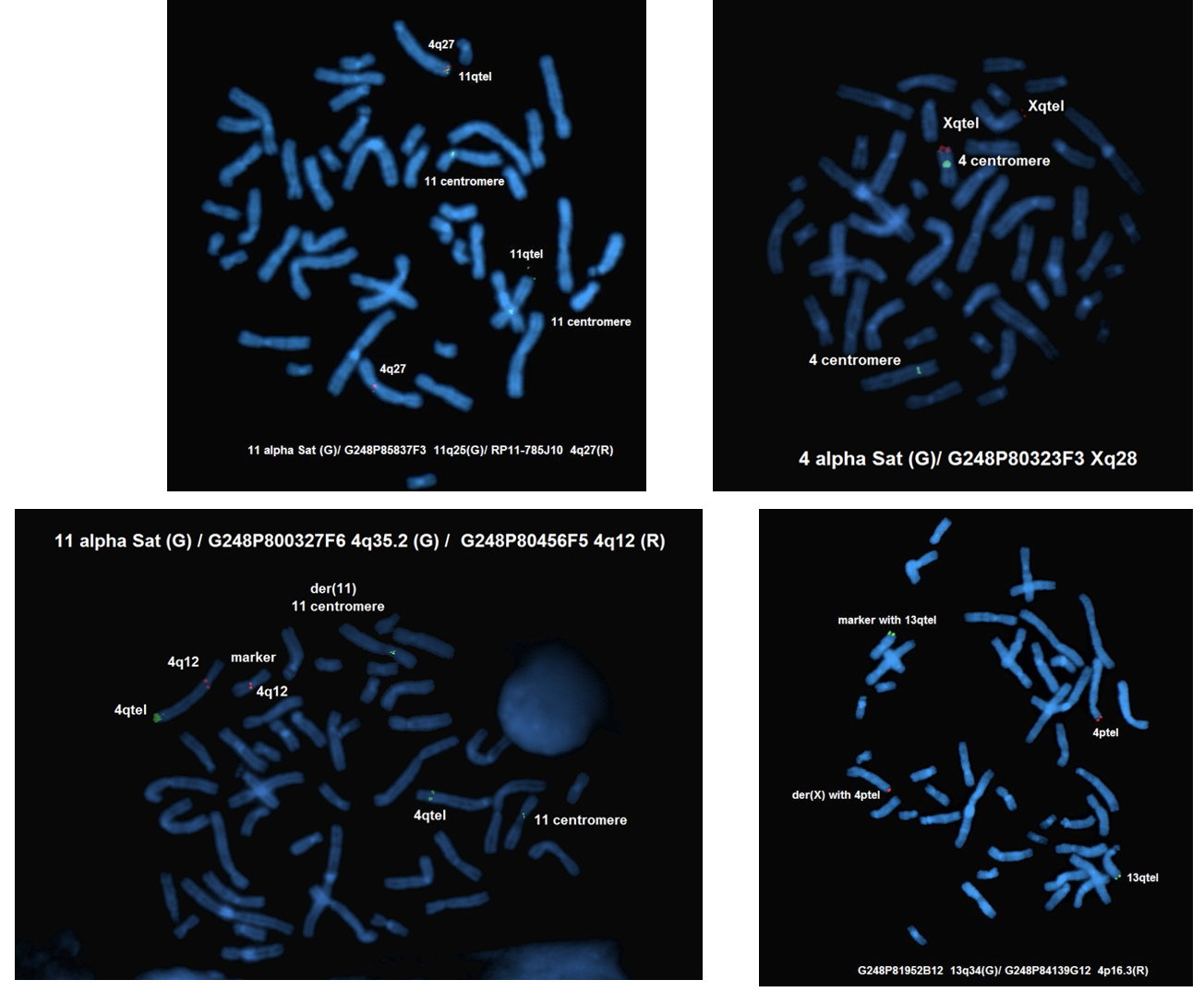


**Figure S2. Fluorescence in situ hybridization (FISH) images for rearranged chromosomes.** FISH probes include G248P84139G12 (4p16.3), G248P80456F5 (4q12), RP11-785J10 (4q27), G248P800327F6 (4q35.2), G248P85837F3 (11q25), G248P81952B12 (13q34), G248P80323F3 (Xq28/Yq12), and centromere probes (alpha satellite) for chromosomes 4 and 11.

**S3**


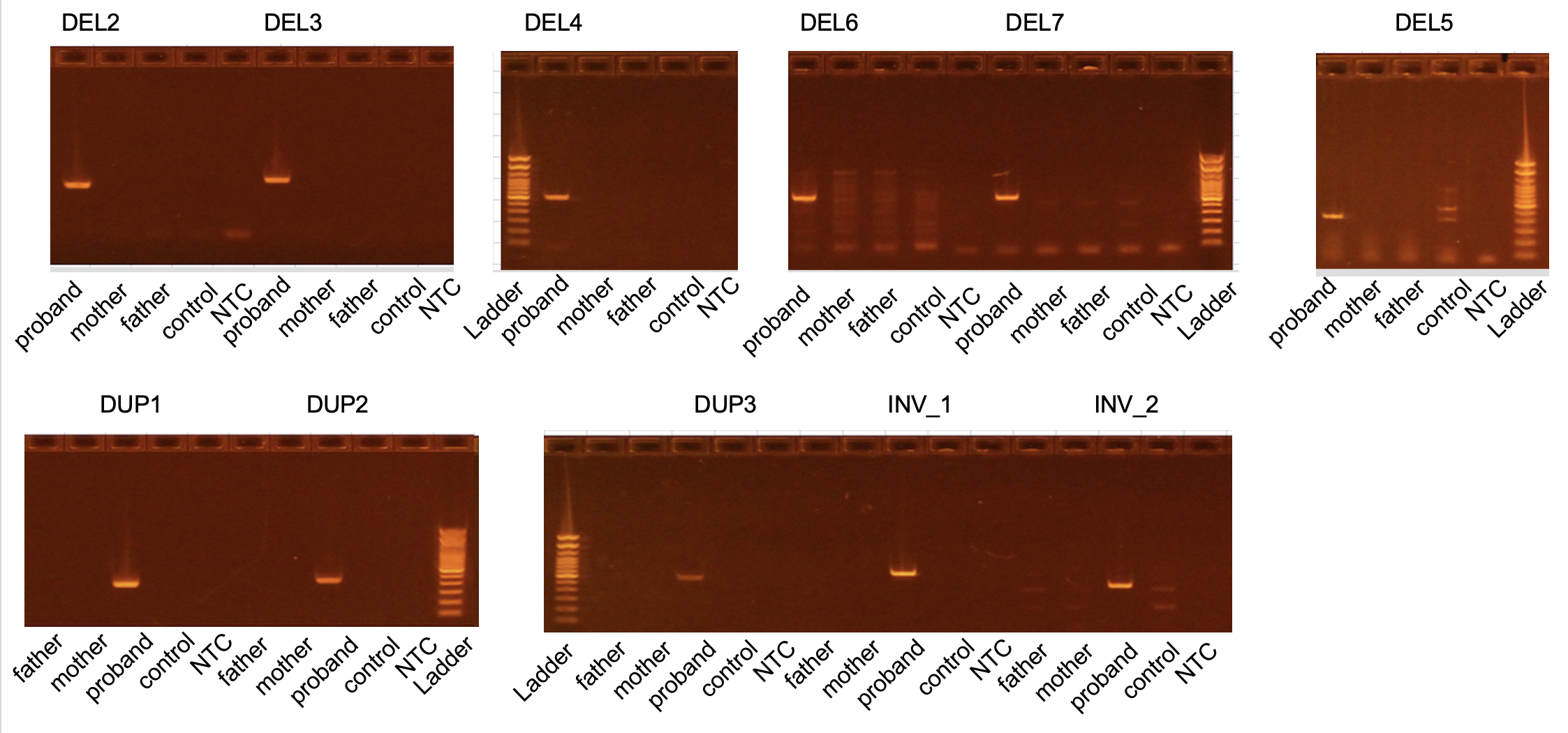


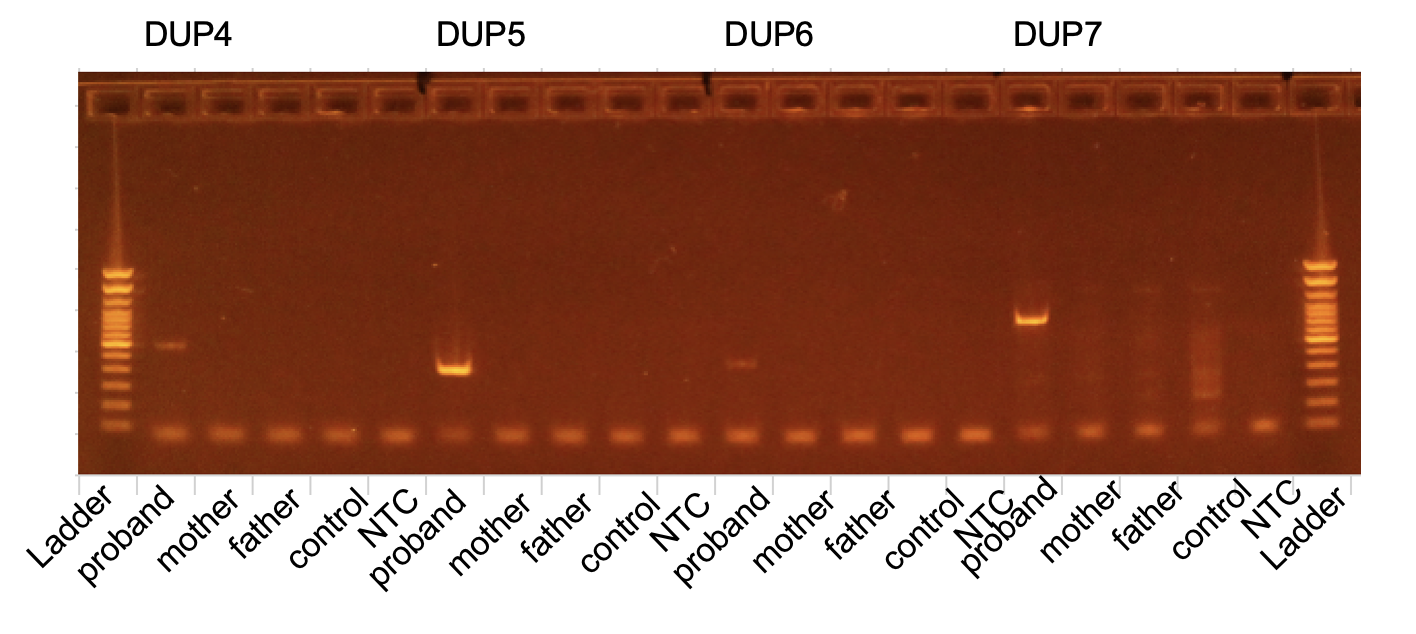


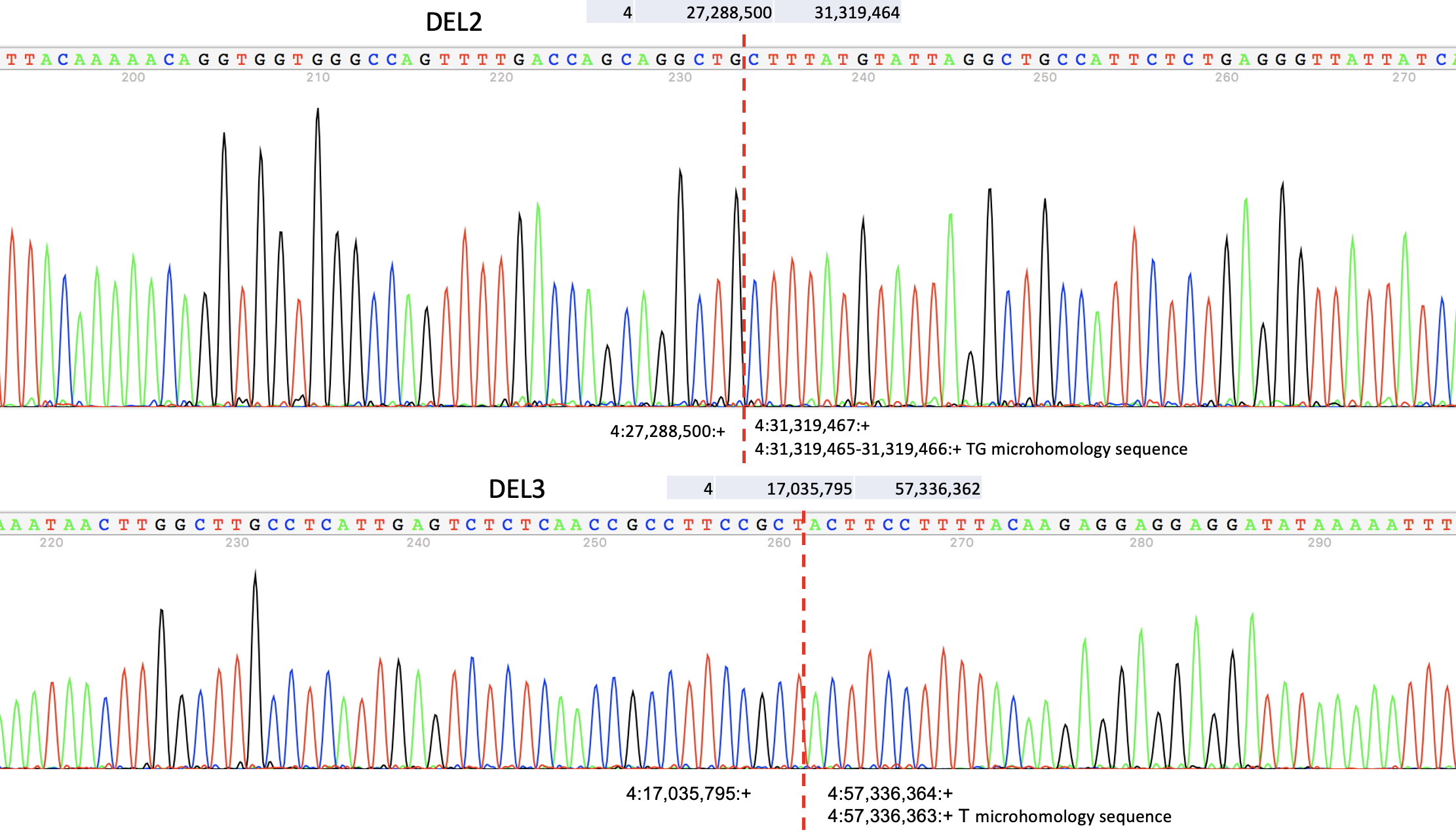


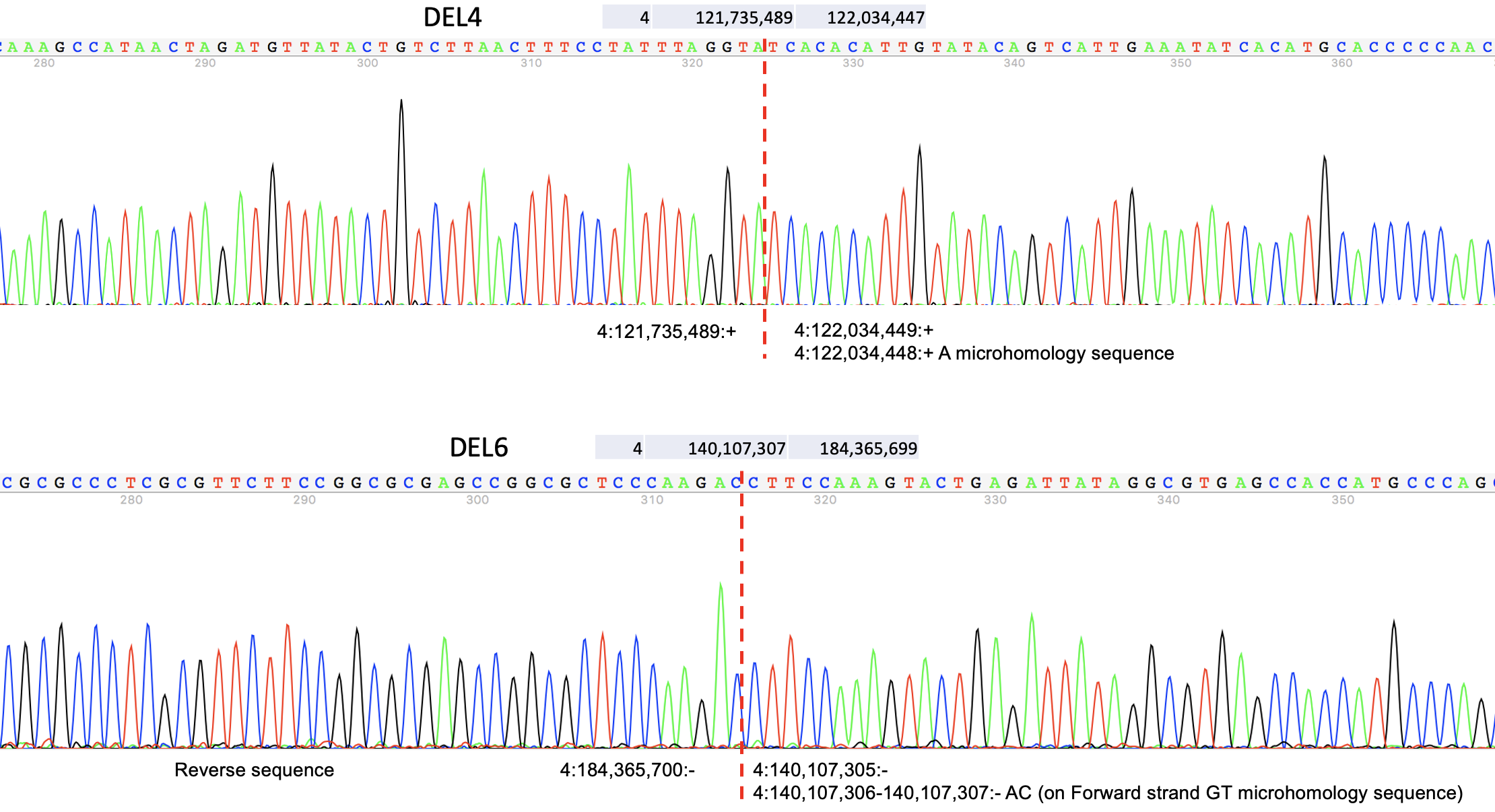


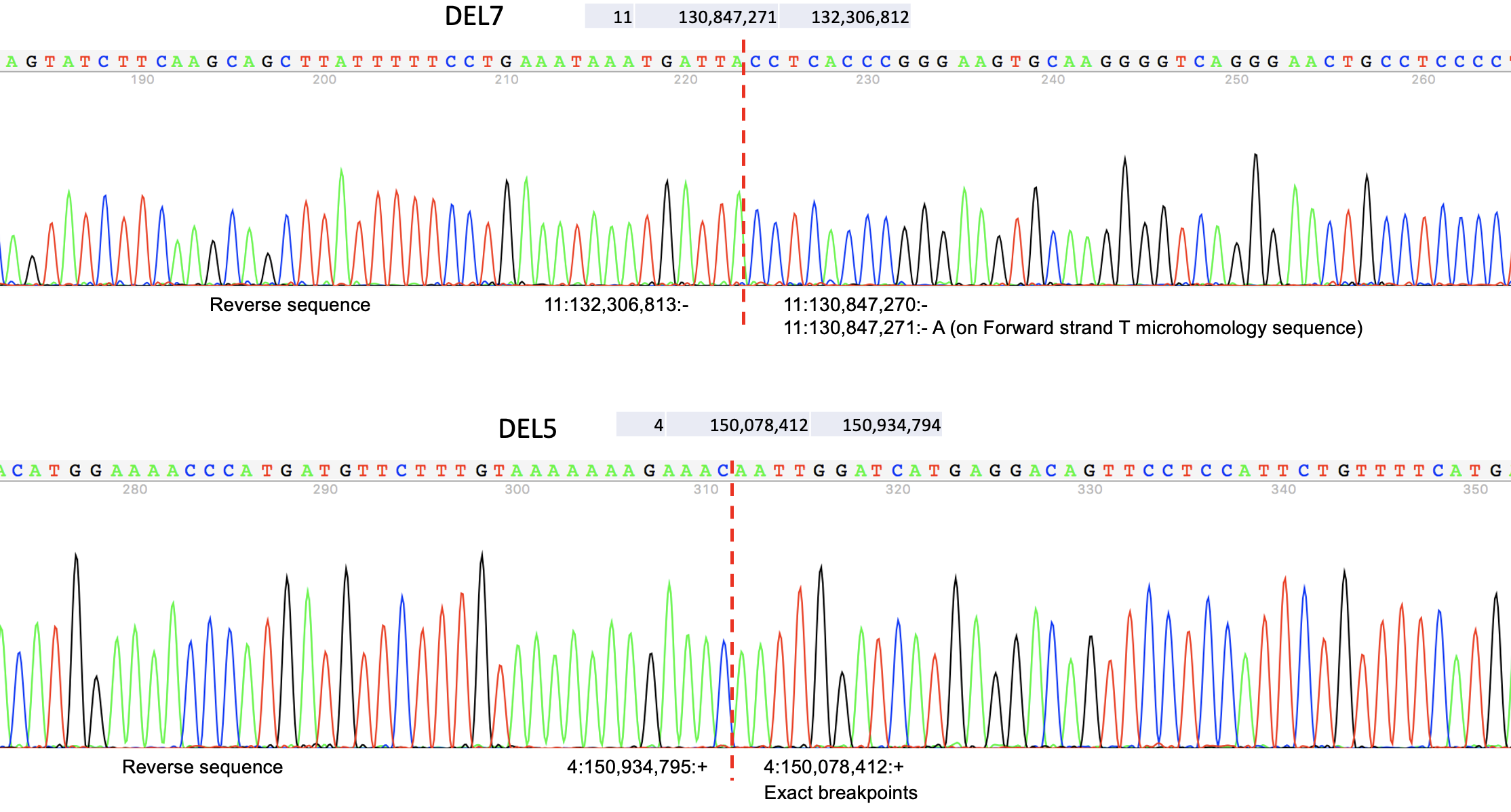


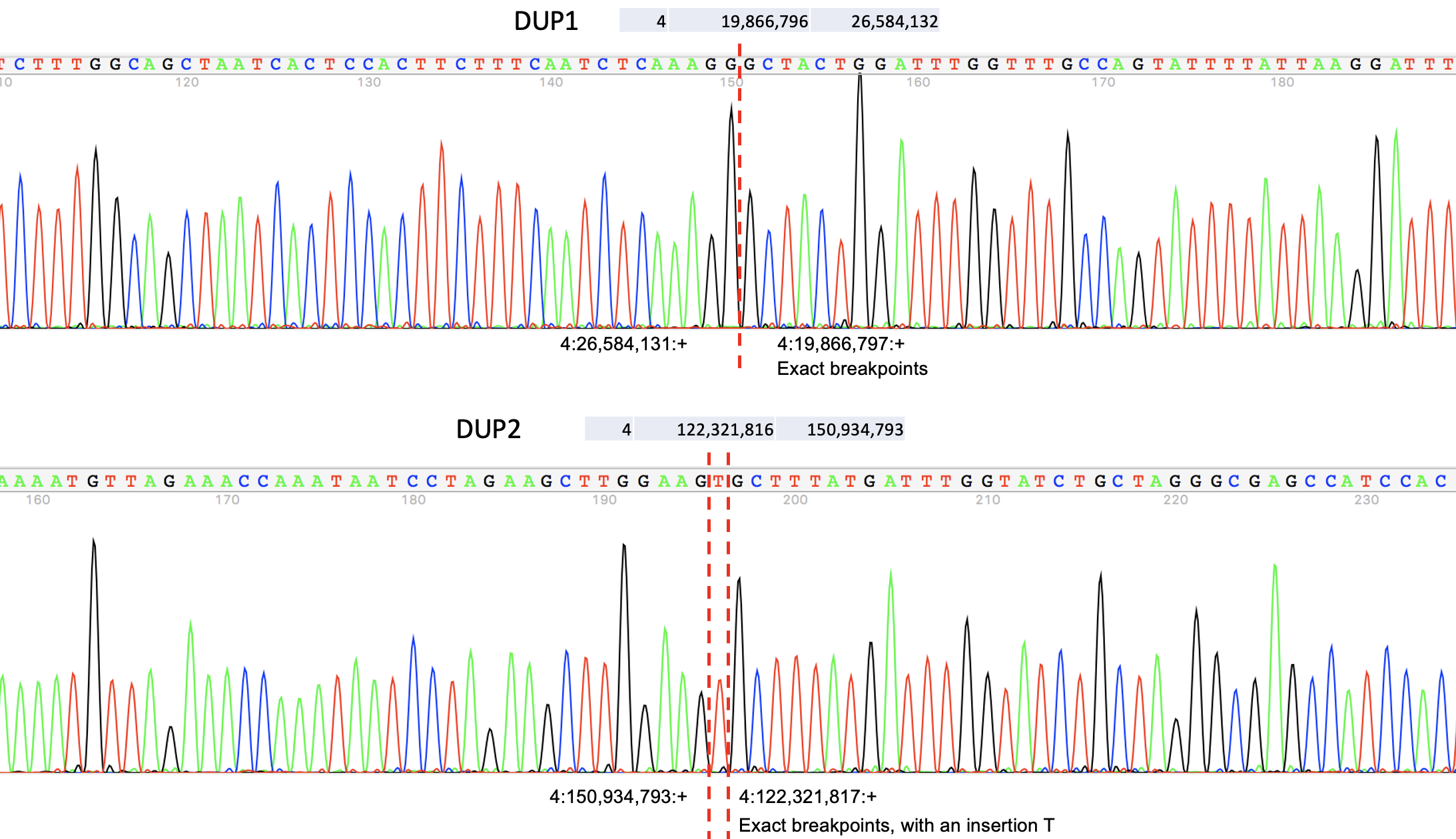


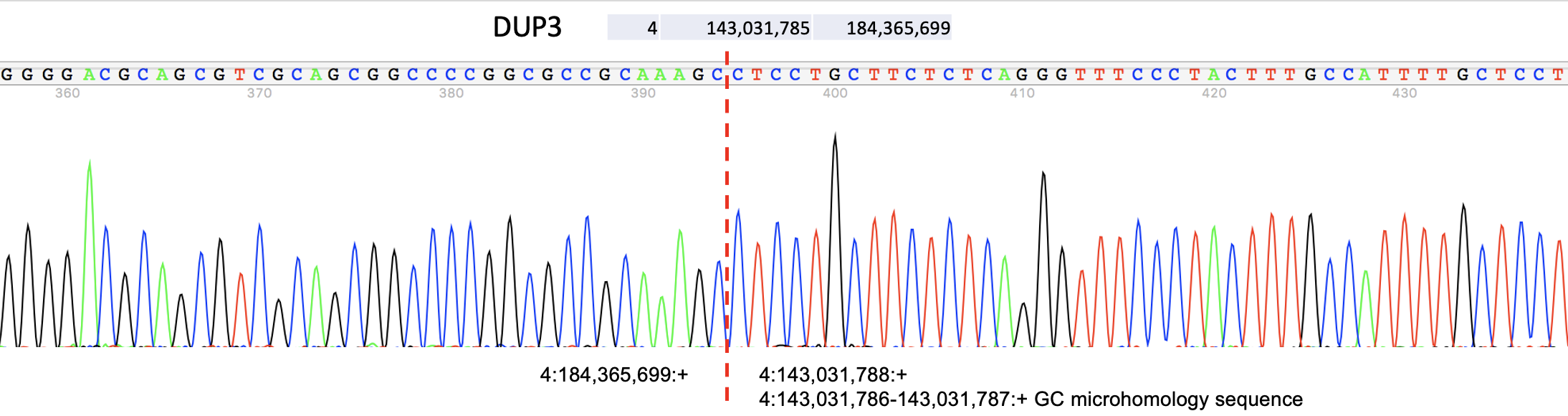


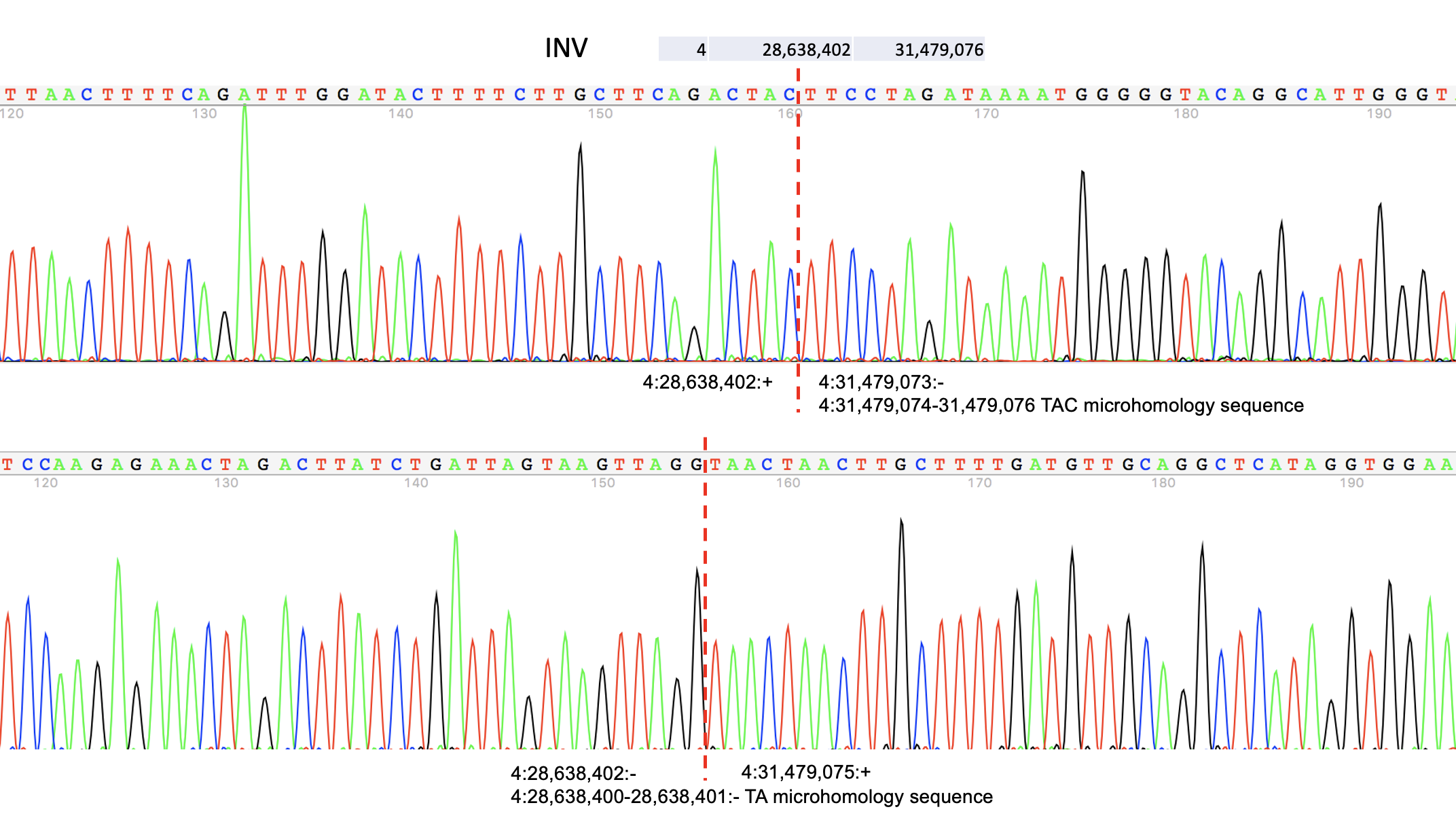


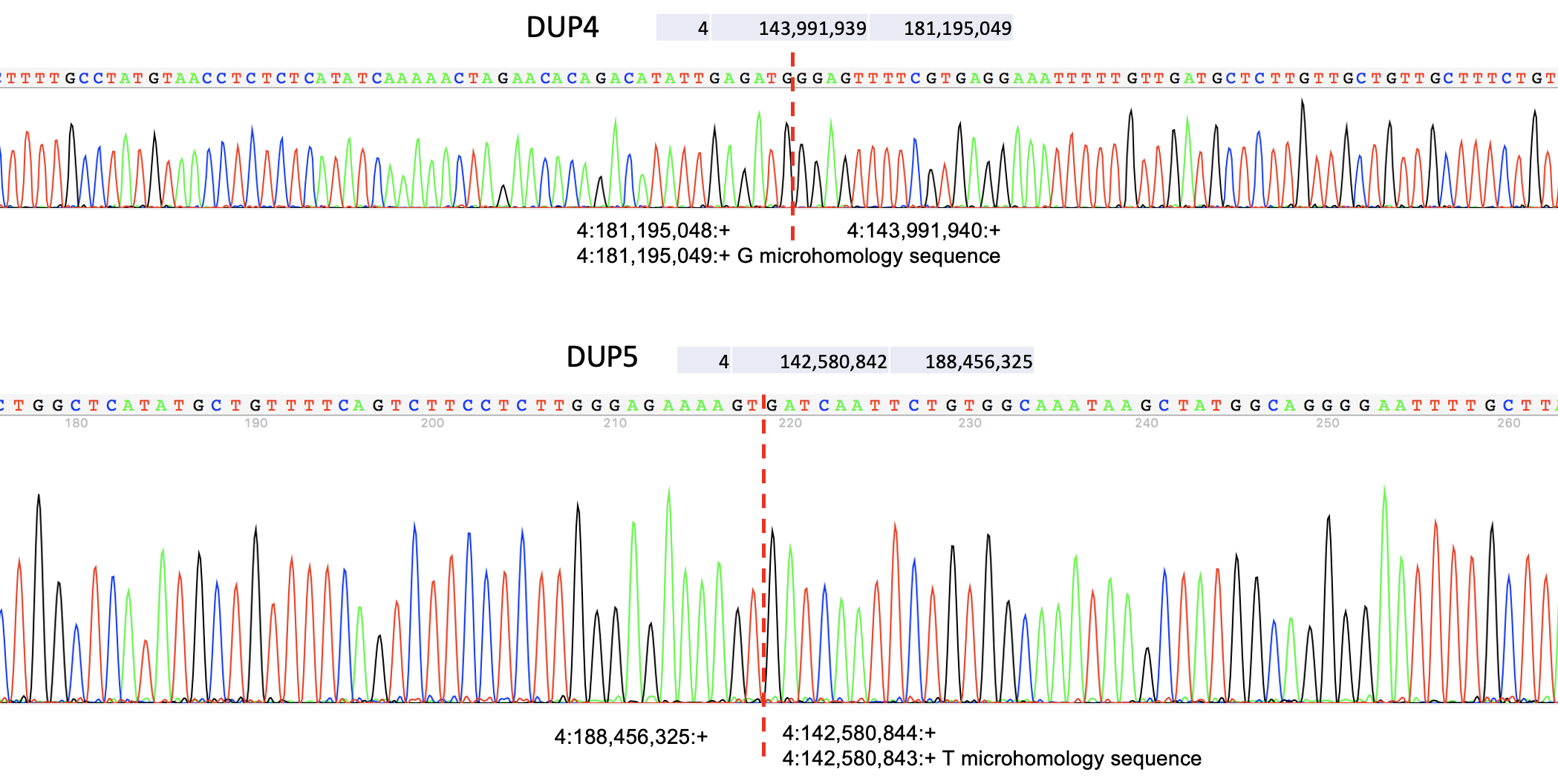


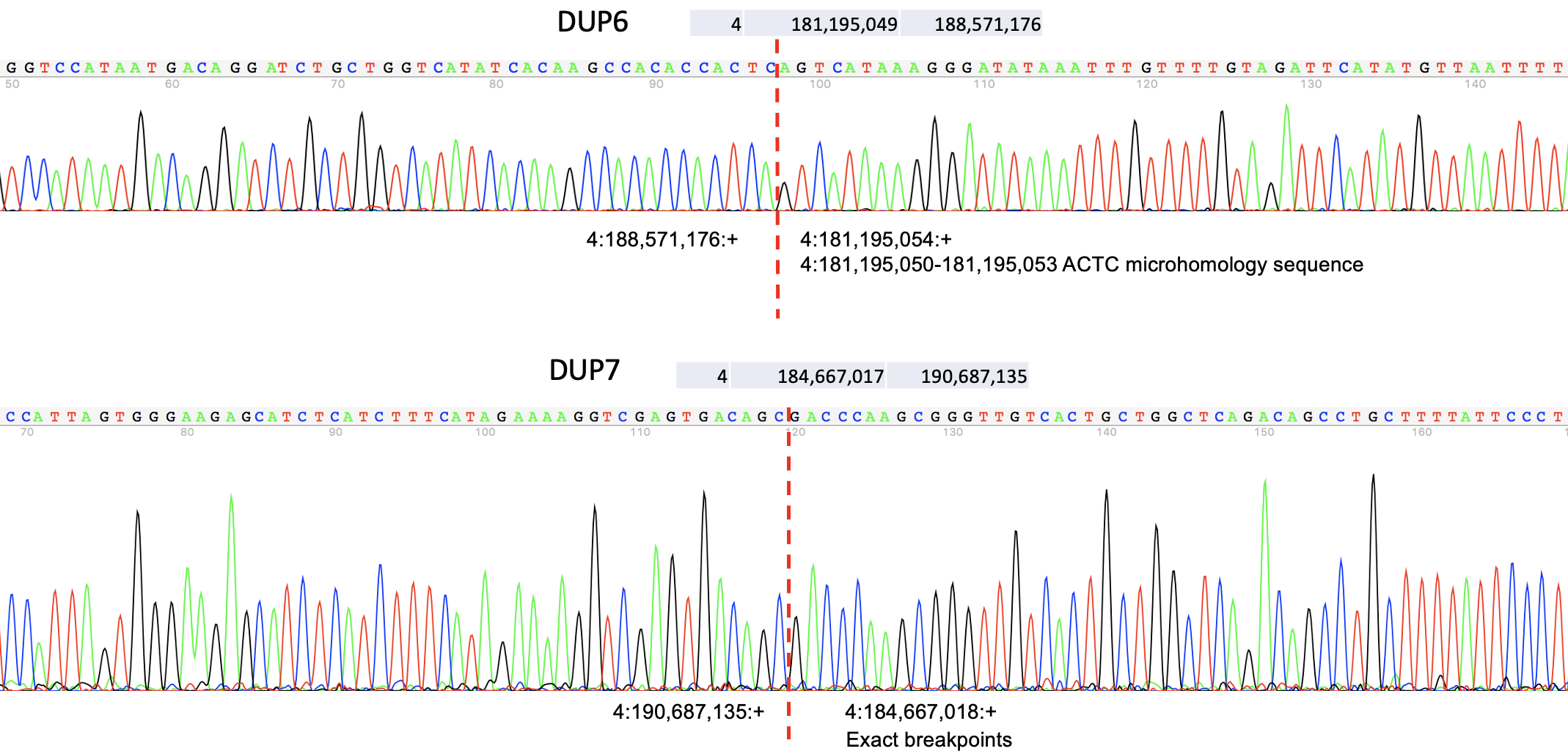


**Translocations**


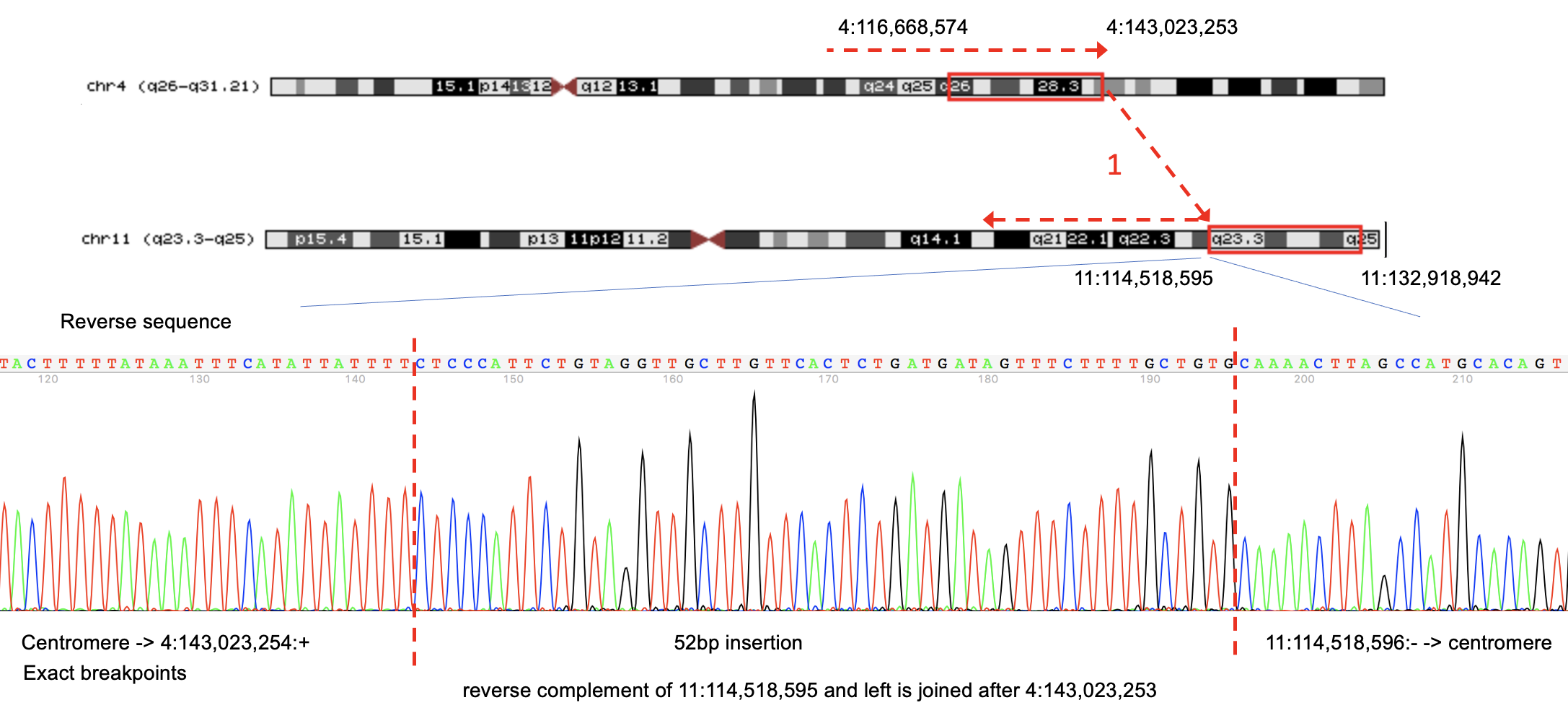


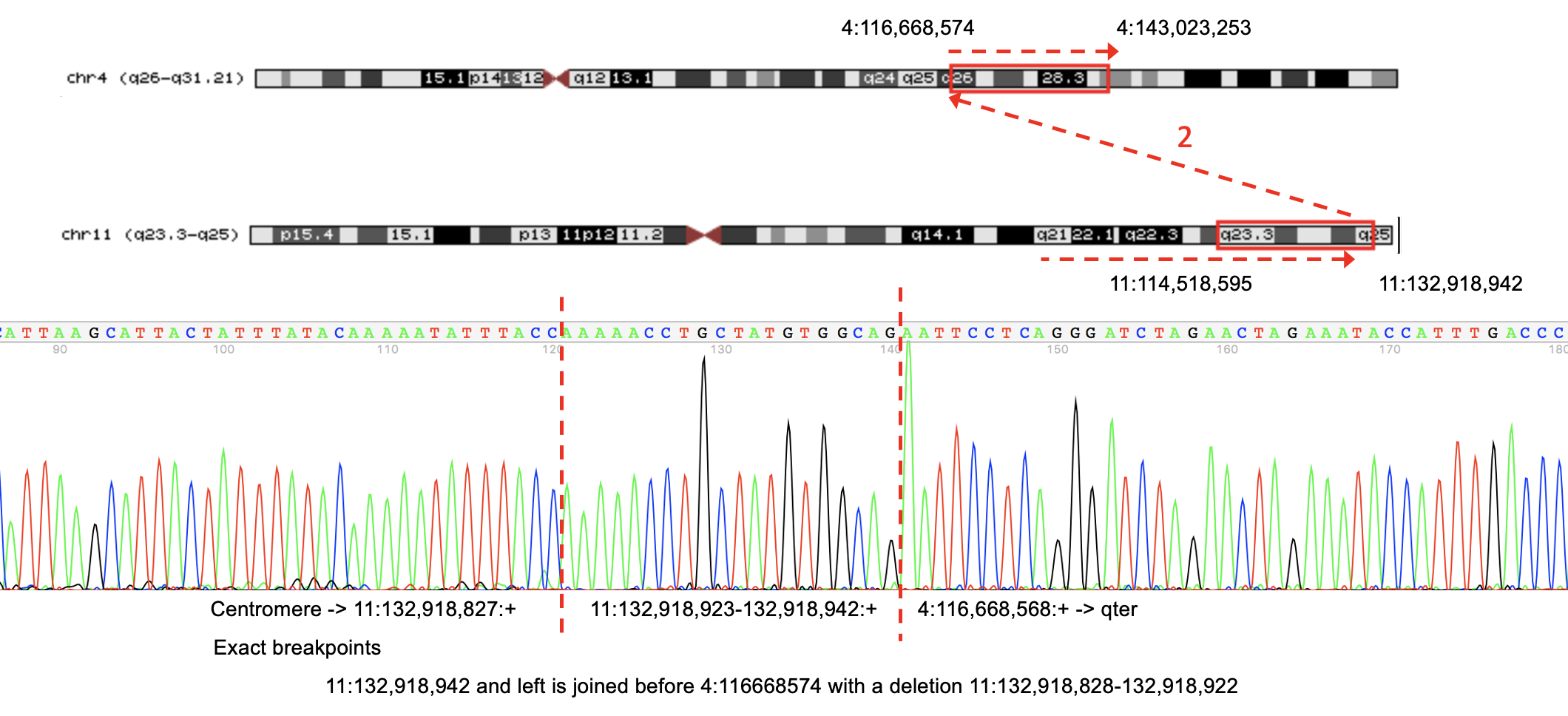


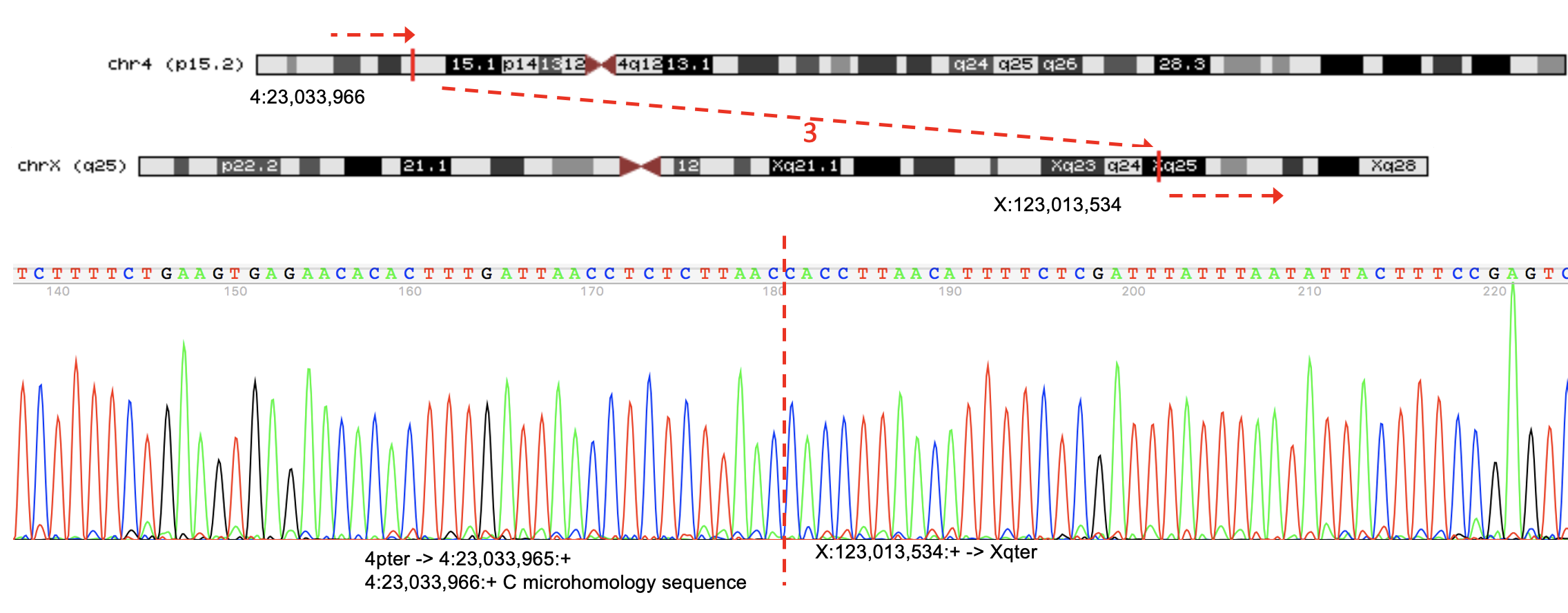


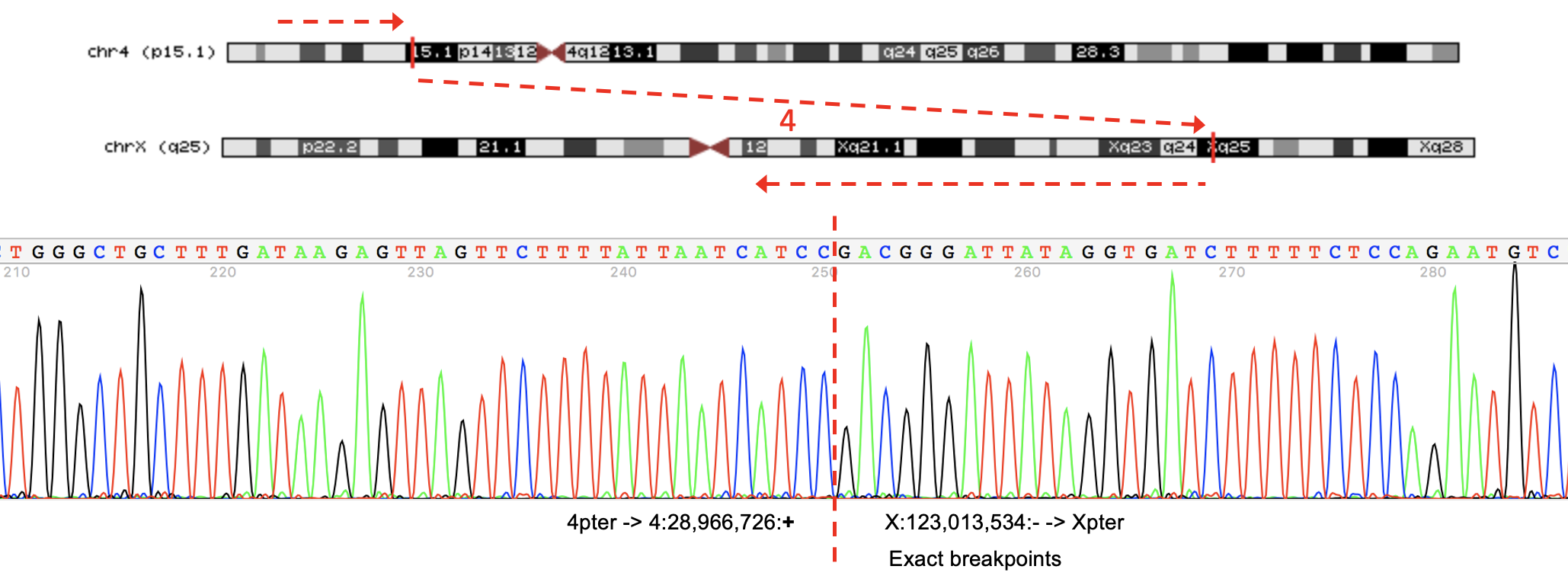


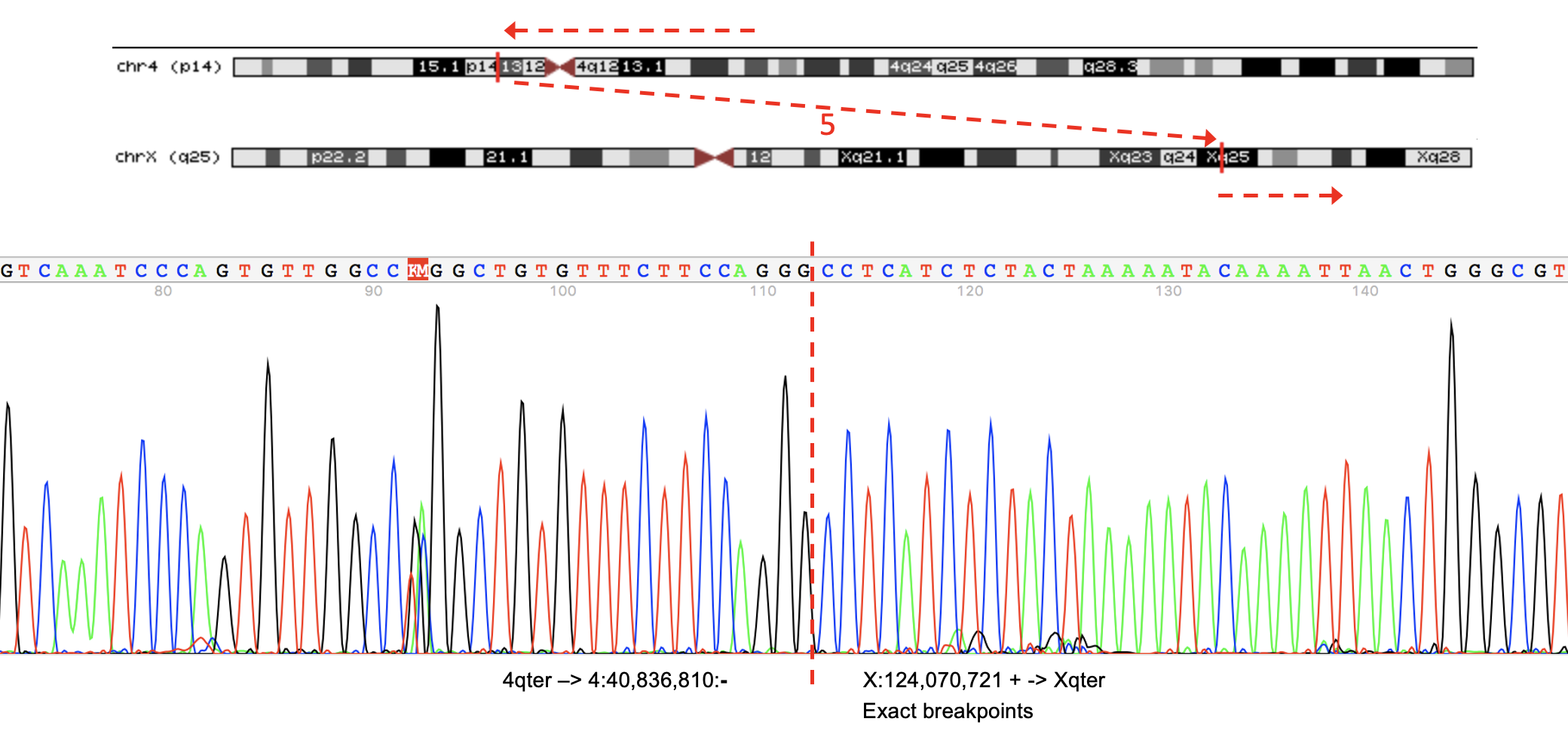


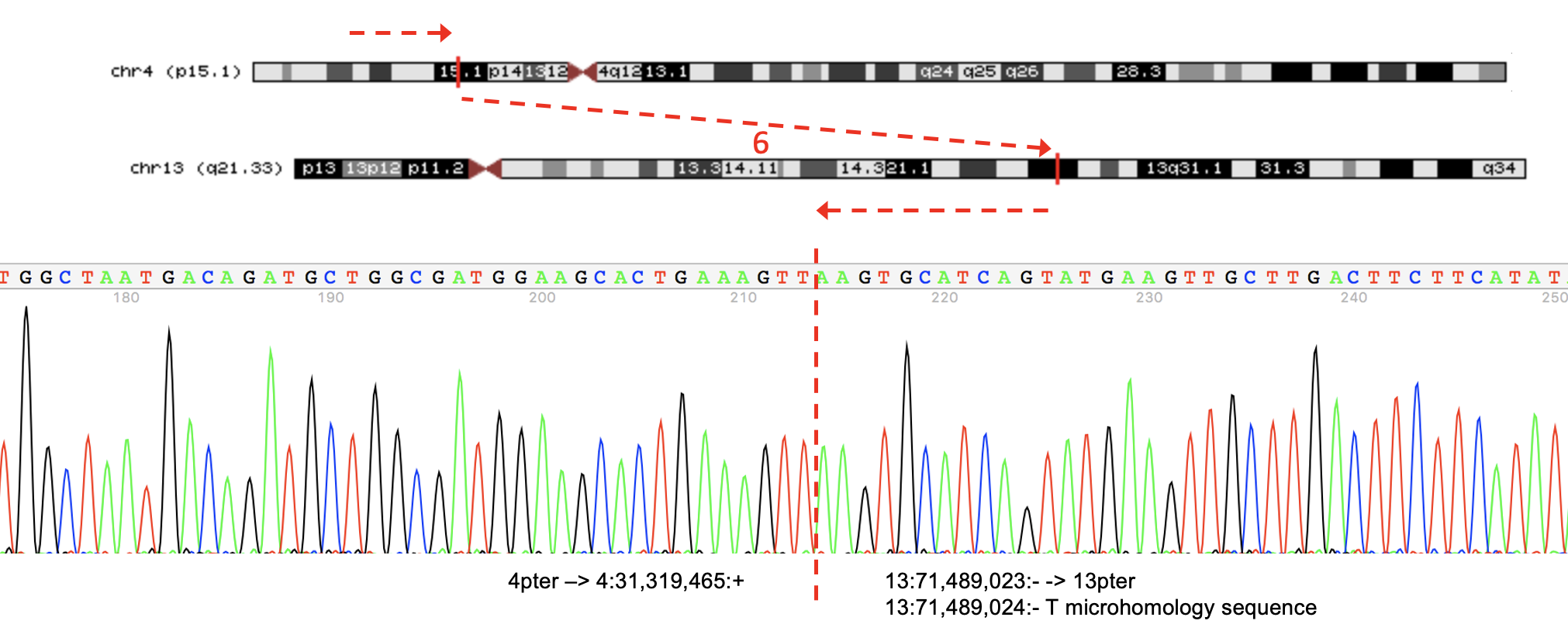


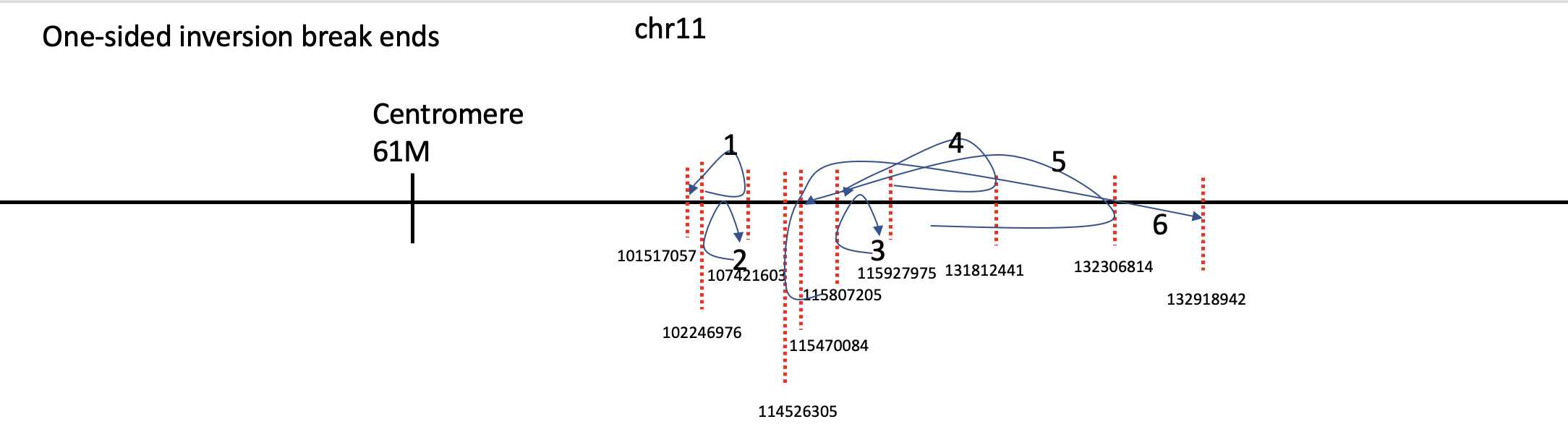


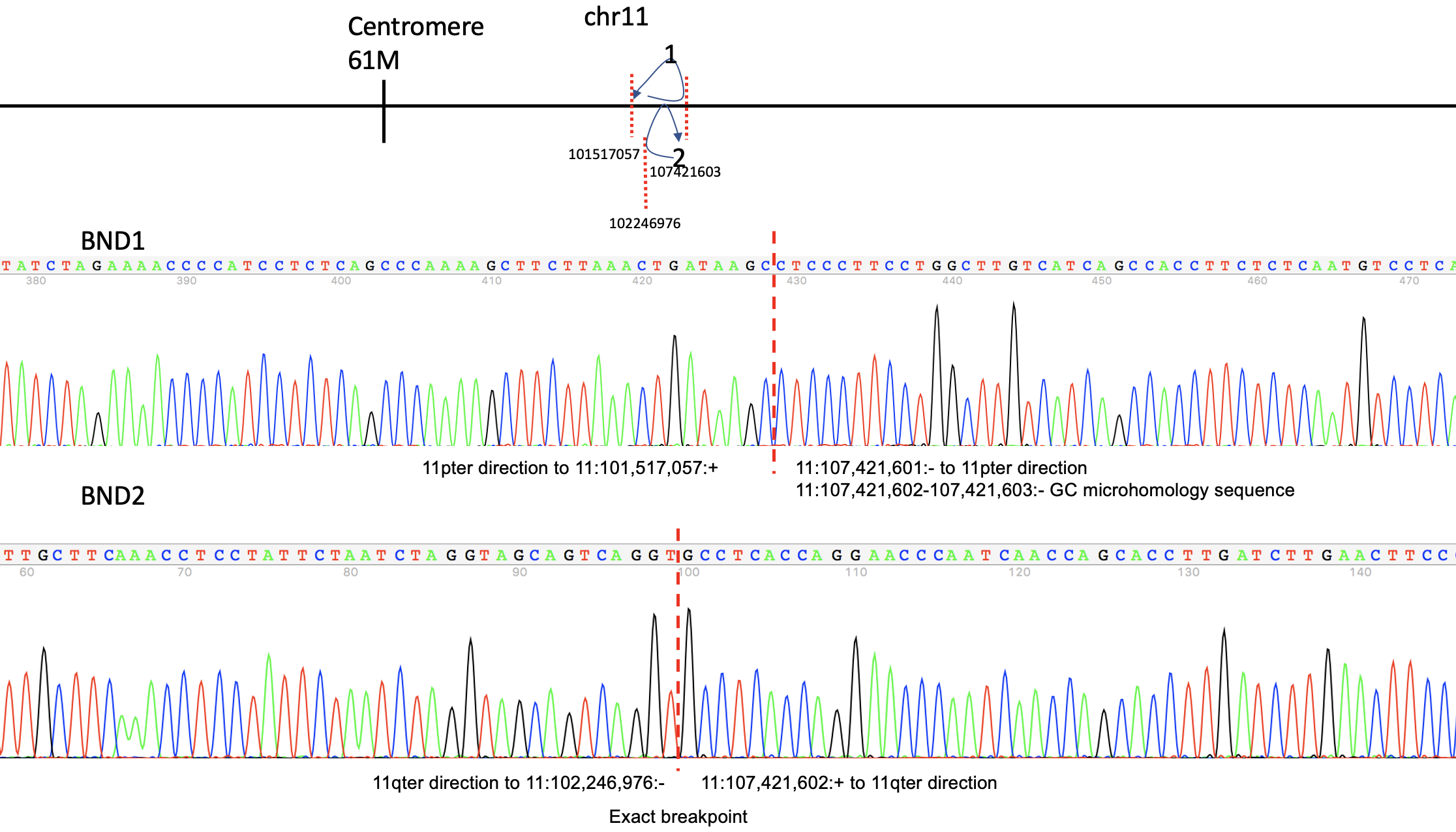


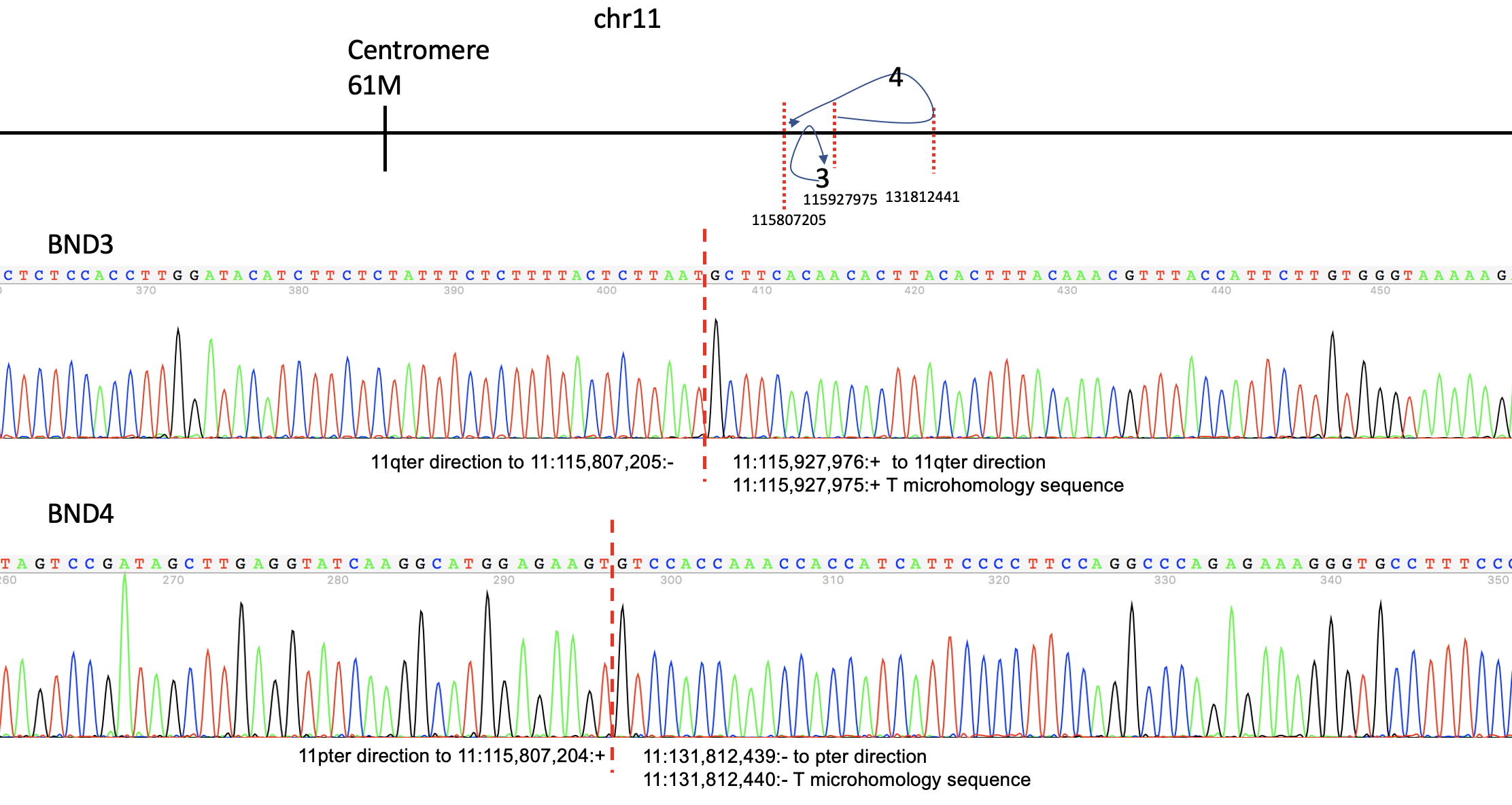


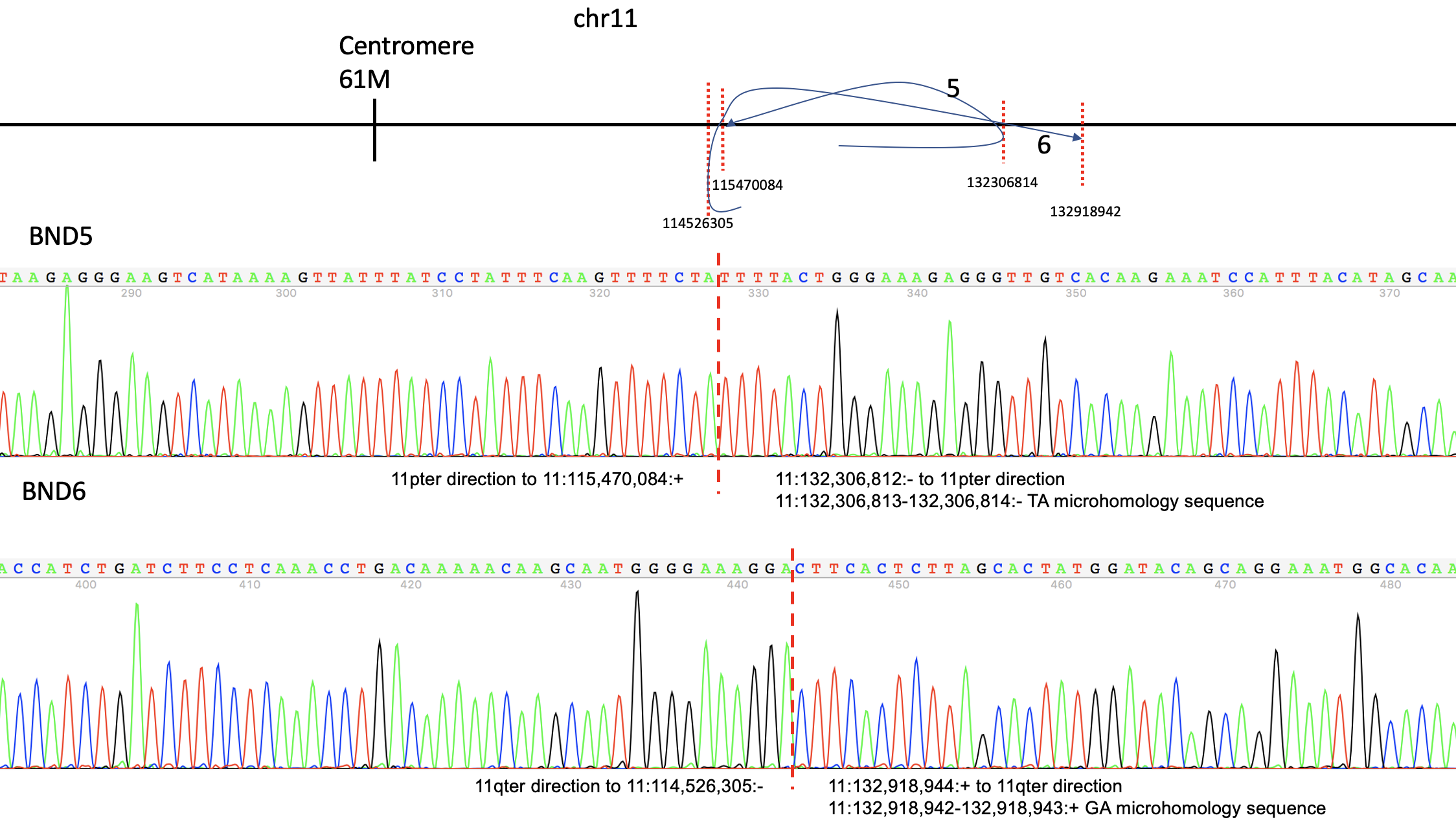


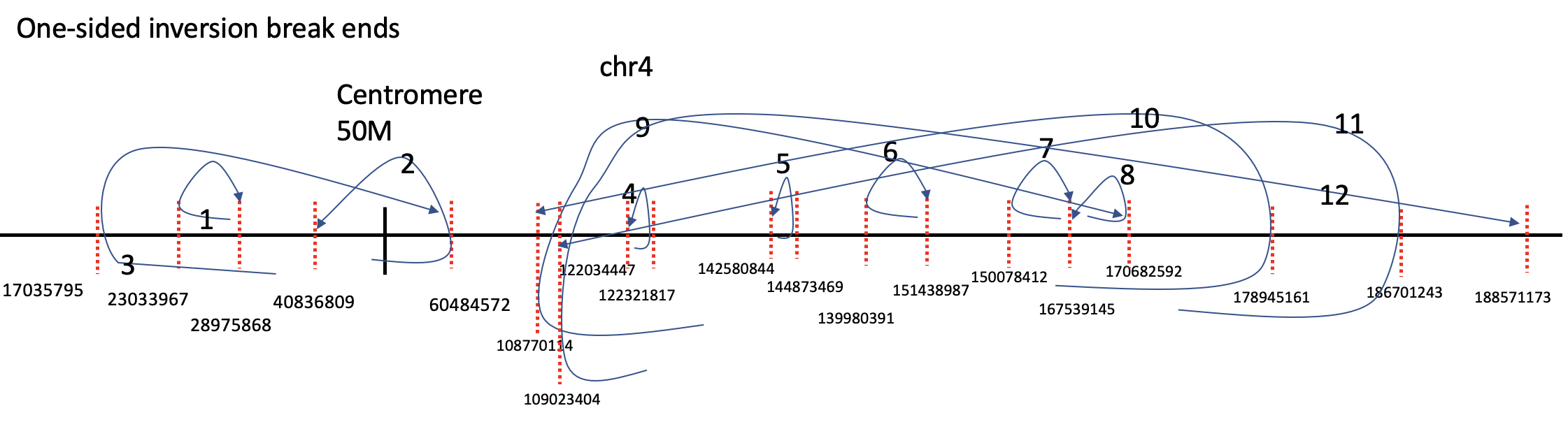


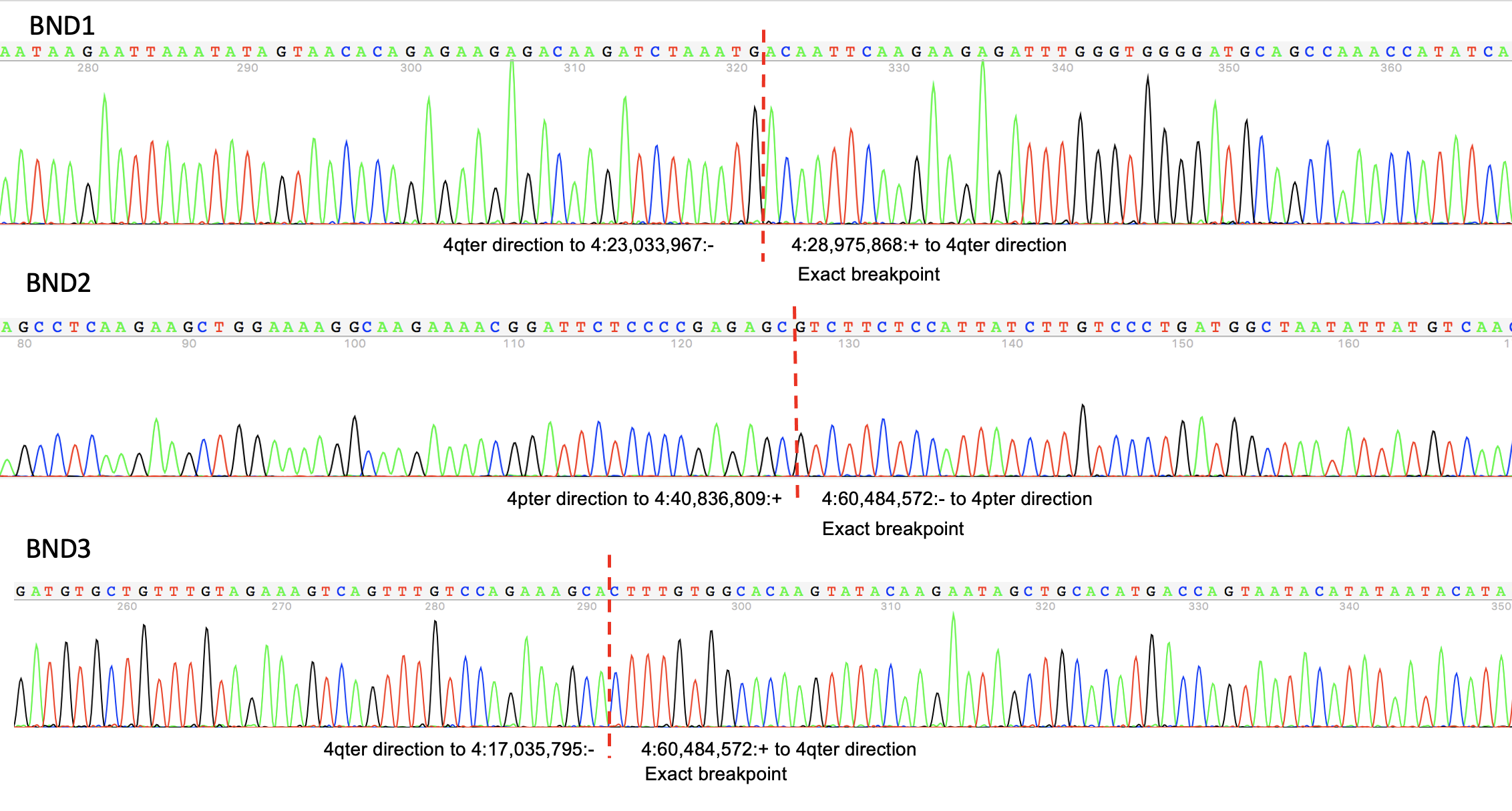


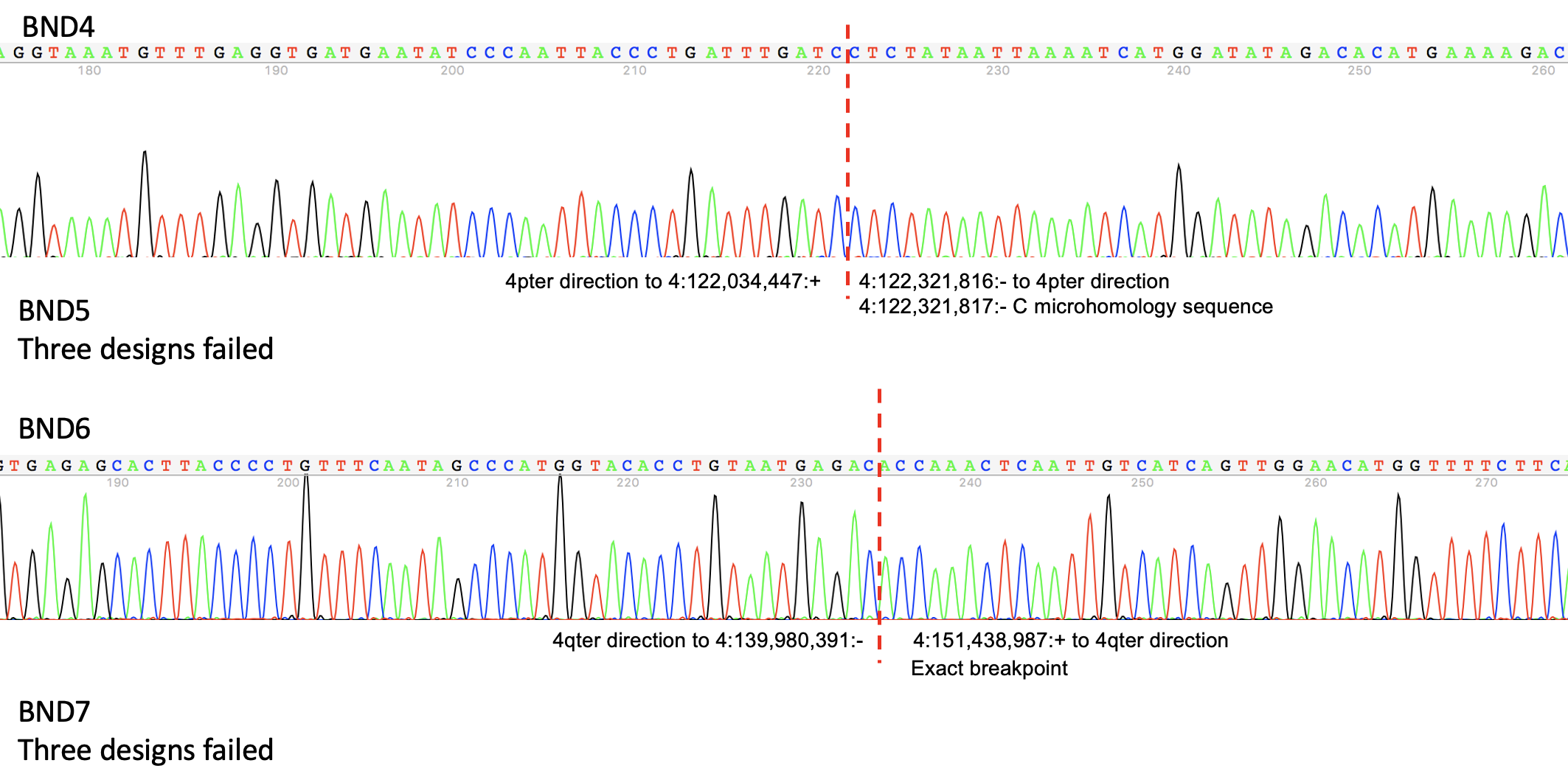


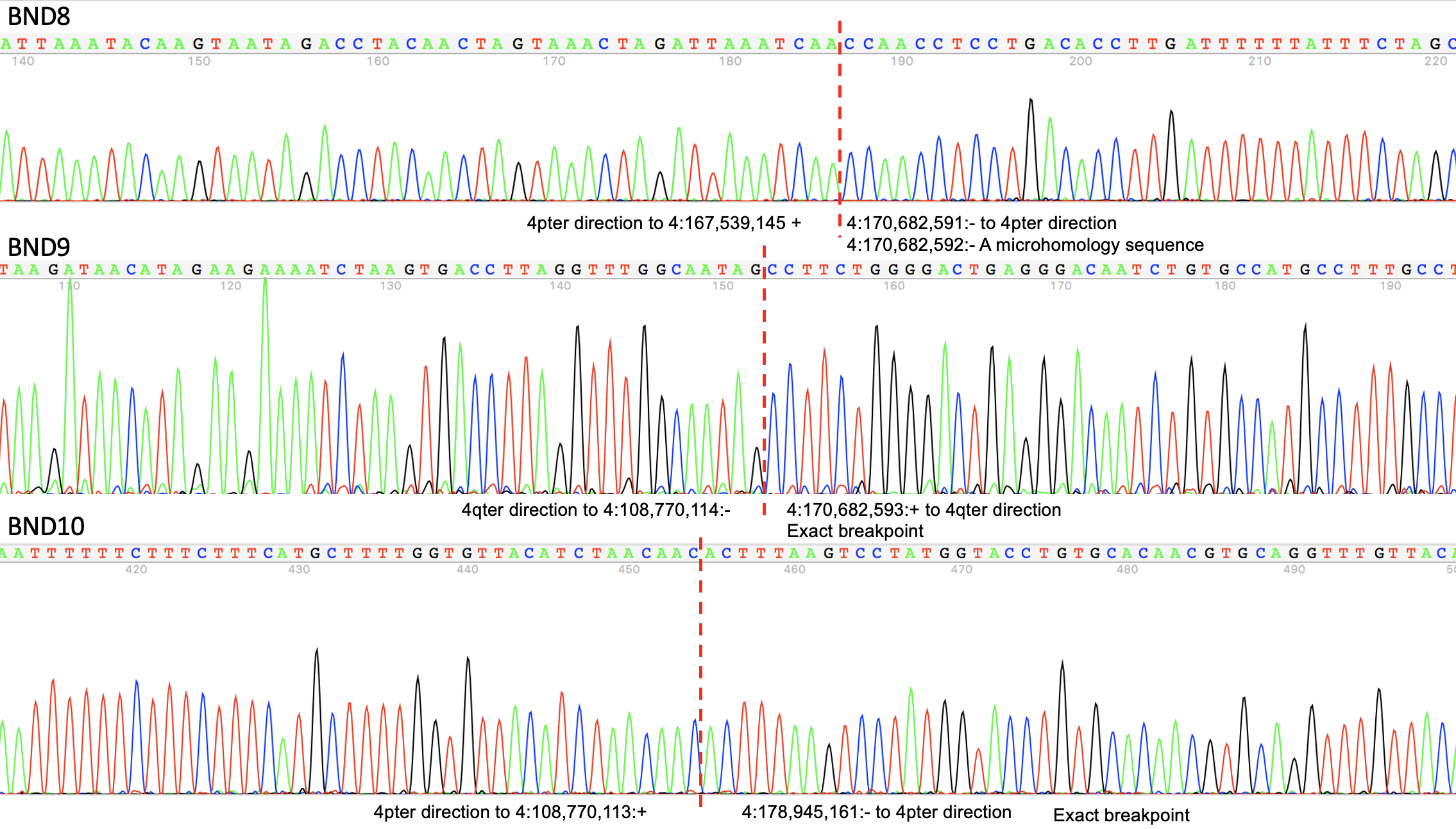


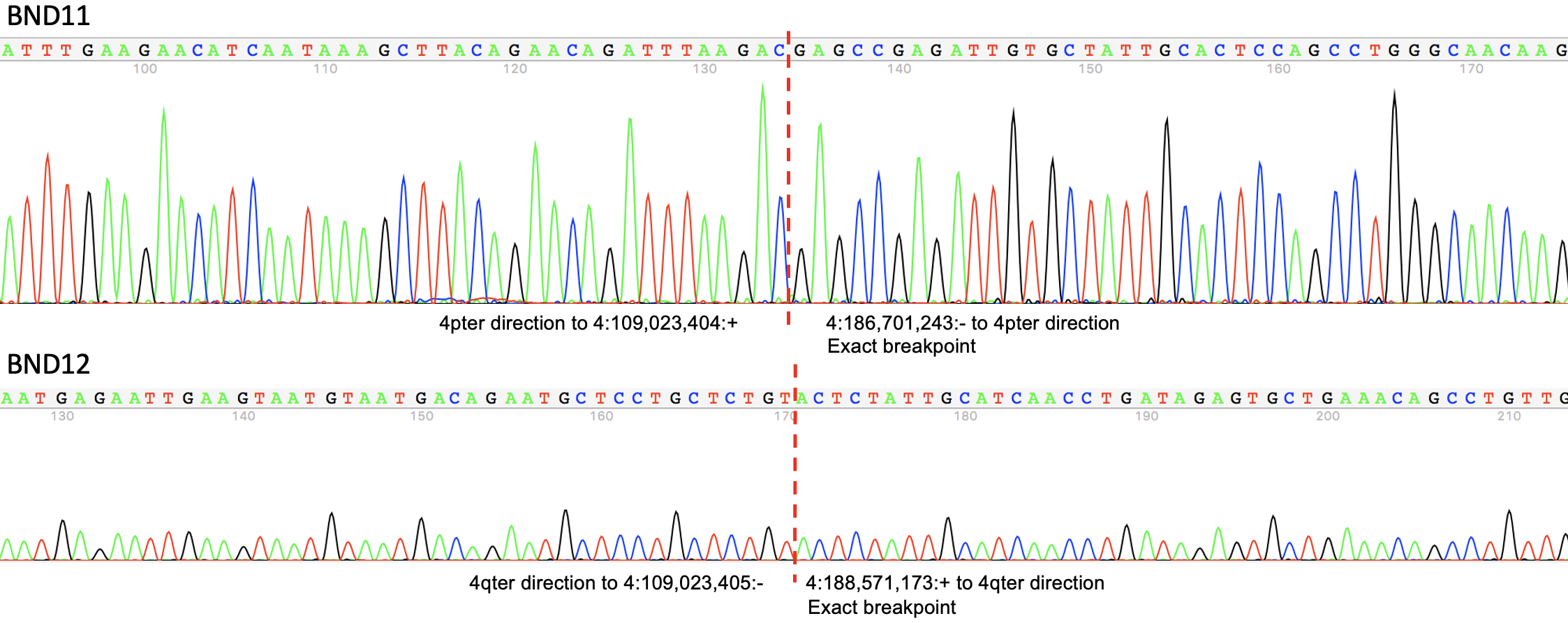


**Figure S3.** Breakpoint mapping of complex rearrangements by PCR and Sanger sequencing. 30 out of 32 putative structural rearrangement events were confirmed at the nucleotide level with patient-specific PCR products.
